# Crucial roles of CircRNA-mediated autophagy in digestive cancer

**DOI:** 10.3389/fonc.2025.1615306

**Published:** 2025-08-27

**Authors:** Shenggan Shi, Ailing Wei, Yong Yang, Juan Li

**Affiliations:** ^1^ Department of Pharmacy, Chengdu Wenjiang District People’s Hospital, Chengdu, Sichuan, China; ^2^ Department of Pharmacy, Personalized Drug Research and Therapy Key Laboratory of Sichuan Province, Sichuan Provincial People’s Hospital, School of Medicine, University of Electronic Science and Technology of China, Chengdu, Sichuan, China

**Keywords:** digestive cancer, circular RNA, autophagy, molecular mechanisms, chemoresistance

## Abstract

Digestive cancers are a significant global public health challenge due to their high incidence and mortality rates. Emerging research highlights the pivotal role of circular RNA (circRNA) and autophagy in the progression of digestive cancers. This review provides a summary of recent findings, showing that circRNA-mediated autophagy could either promote or suppress tumor development in esophageal, gastric, liver, pancreatic, and colorectal cancers. CircRNAs could regulate autophagy in digestive cancers by acting as competitive endogenous RNAs, influencing downstream target genes and signaling pathways. Dysregulated circRNAs contribute to tumor onset, progression, and chemoresistance by altering autophagy levels. Targeting specific circRNAs to modulate autophagy may offer potential as a diagnostic, prognostic biomarker, or therapeutic strategy for digestive cancers.

## 1 Introduction

Digestive cancers include esophageal squamous cell carcinoma, gastric cancer, colorectal cancer, hepatocellular carcinoma, pancreatic cancer and related cancers (1). These cancers contribute to approximately one-third of global cancer-related deaths (2). Among digestive cancers, colorectal, gastric, and liver cancers are the most prevalent globally. In 2022, nearly 20 million new cancer cases were reported worldwide, including 1.9 million cases of colorectal cancer (9.6% of all cases) and 0.98 million cases of gastric cancer (4.9%). Colorectal cancer ranked as the second leading cause of cancer deaths (9.3%), followed by liver cancer (7.8%) and gastric cancer (6.8%) (3, 4). As the global population ages, the incidence of digestive cancers is projected to rise significantly, imposing substantial economic challenges (5). By 2050, the global treatment costs for digestive tract cancers are estimated to reach $5.86 trillion (6). This underscores the urgent need for prioritized screening and interventions to mitigate future economic impacts. Despite technological advancements in diagnosing and treating digestive system cancers, these diseases remain a leading cause of death, particularly in patients with advanced or metastatic conditions. Surgery remains the primary treatment for early-stage digestive cancers. However, advanced tumors often resist chemotherapy and radiotherapy due to cancer cell spread, resulting in limited treatment efficacy and poor patient prognosis. Understanding the molecular mechanisms underlying digestive system cancer is essential for advancing diagnosis, treatment, and prognosis. Early detection of digestive system cancer and improving its sensitivity to radiotherapy and chemotherapy remain critical challenges.

**Figure 1:**
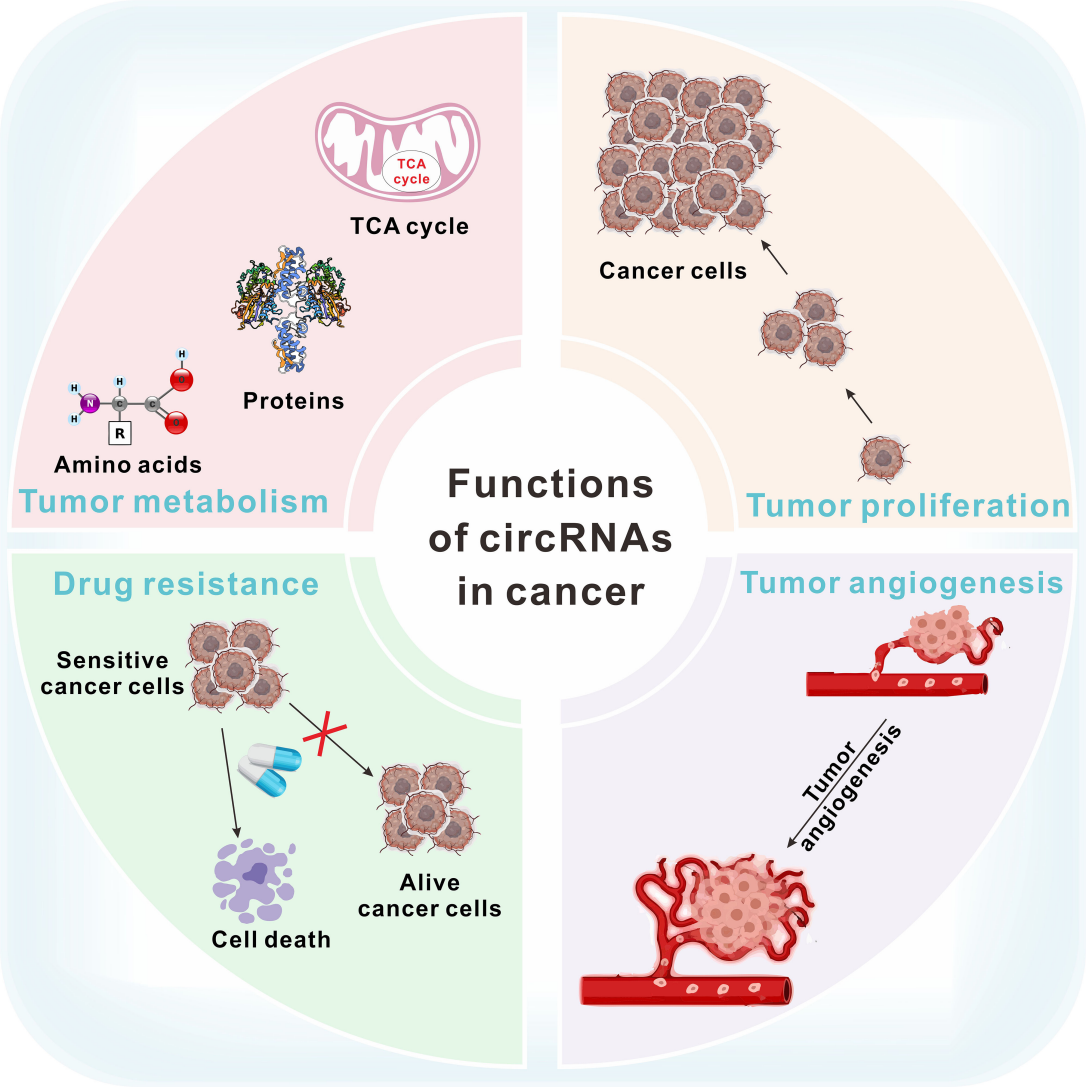
The function of circRNAs in tumor metabolism, proliferation, drug resistance and angiogenesis.

Circular RNAs (CircRNAs) are non-coding RNA molecules that lack 5′-caps and 3′-polyadenylated tails, forming a stable circular structure through covalent bonds (7, 8). Initially, circRNAs were thought to be by-products of abnormal splicing (9). However, recent advances in research have revealed their significant roles in cancer, cardiovascular diseases, and chemoresistance (10–12). The function of circRNAs in tumors is shown in **Figure 1**. CircRNAs have dual effects on cancer progression. Cancer-related circRNAs are classified as oncogenic circRNAs and tumor-suppressive circRNAs (13). Oncogenic circRNAs promote tumor progression and are typically overexpressed in cancer cells. Tumor-suppressive circRNAs prevent tumorigenesis by regulating processes like cell growth, apoptosis, and other cancer-related activities (14). Du et al. found that m6A-mediated upregulation of circMDK contributed to tumorigenesis in lung cancer (15). Wang et al. reported that upregulation of hsa_circRNA_100269 suppressed gastric cancer growth and metastasis by inactivating the PI3K/Akt axis (16).

**Figure 2:**
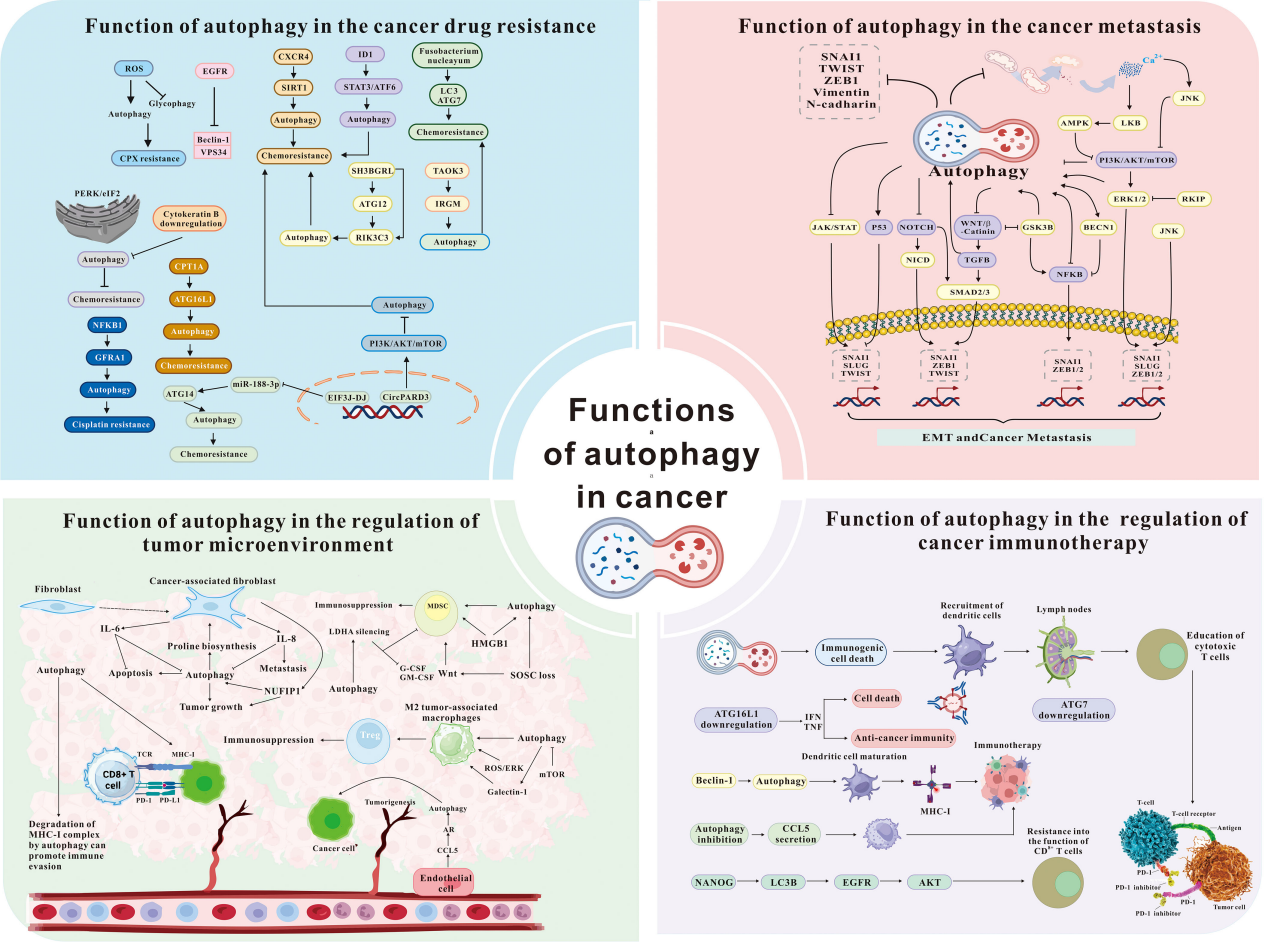
The role of autophagy in cancer drug resistance, metastasis, microenvironment, and immunotherapy [modified from Zada et al. (17) and Niu et al. (18)]. ROS, reactive oxygen species; EGFR, epidermal growth factor receptor; CXCR4, C-X-C chemokine receptor 4; SIRT1, sirtuins1; ID1, inhibitor of DNA binding 1; STAT3, signal transducer and activator of transcription 3; ATF6, activating transcription factor 6; LC3, microtubule-associated protein light chain 3; ATG, autophagy related; TAOK3, TAO kinase 3; IRGM, immunity related GTPase M; NFKB1, nuclear factor kappa B subunit 1; JAK, janus kinase; TGFB, TGF-beta; SMAD2/3, SMAD family member 2/3; LKB, liver kinase B; RKIP, Raf kinase inhibitory protein; GSK3B, glycogen synthase kinase 3 Beta; NANOG, nanog homeobox; IFN, interferon; TNF, tumor necrosis factor; IL-6, interleukin-6; IL-8, interleukin-8; NUFIP1, nuclear fragile X mental retardation-interacting protein 1; HMGB1, high mobility group box 1; CCL5, C-C motif chemokine ligand 5.

Research highlights autophagy as a key factor in the progression of various cancers (**Figure 2**), including those affecting the digestive system (19). Autophagy contributes to cancer progression by enabling survival under nutrient deprivation and hypoxia (20, 21), while also inducing programmed cell death to suppress cancer growth (22). Growing evidence suggests that circRNA promotes apoptosis by regulating autophagy (23). CircRNAs regulate autophagy through multiple mechanisms including regulating the expression of autophagy-related genes and proteins (24–26). Furthermore, circRNAs function as molecular sponges, binding to autophagy-related miRNAs, thereby influencing cancer phenotypes such as survival, proliferation, epithelial-mesenchymal transition, migration, invasion, angiogenesis, and metastasis (25, 27–30). For example, (1) Initiation phase: CircRNAs modulated autophagy onset by influencing the PI3K/AKT/mTOR signaling pathway (31); (2) Phagophore nucleation stage: Certain circRNAs regulated autophagy initiation by targeting critical molecules such as ATG14 (32) and Beclin1 (32, 33); (3) Membrane expansion stage: CircRNAs affected autophagosome extension by modulating the ATG5–ATG12–ATG16L complex and the LC3 conjugation system (15, 34, 35); (4) Autophagosome maturation and fusion: In later stages, circRNAs regulated proteins such as STX17 (36) and RAB10 (37), thereby influencing the formation and fusion of autolysosomes. However, the precise mechanisms by which circRNA regulates autophagy to affect digestive system tumors remain unclear, necessitating further research. This review synthesizes current literature to explore the role of the circRNA-autophagy axis in the progression and prognosis of digestive system tumors, offering insights for improved treatments. Furthermore, circRNAs have been recognized as key regulators of autophagy in various cancer types (38). This review aims to explore the role of circRNAs in regulating autophagy in digestive system tumors, with the potential to inform the development of novel therapeutic strategies for these cancers.

## 2 The role of autophagy in digestive cancer

Autophagy, also known as type II programmed cell death, is a conserved cellular process that helps cells adapt to external environmental changes while maintaining energy balance and homeostasis (39). The primary forms of autophagy are macroautophagy, microautophagy and molecular chaperone-mediated autophagy (CMA) (40). The most commonly studied autophagy usually refers to macroautophagy. In addition, autophagy also includes selective autophagy such as mitophagy, reticulophagy and ribophagy, etc. Mitophagy, a classical form of selective autophagy, plays a vital role in various cellular processes, including cell survival, differentiation, development, senescence, and apoptosis (41). It is also closely associated with numerous diseases, particularly cancer. Recent studies suggest that mitophagy exerts context-dependent effects during different stages of tumor progression (42): In the early stages of tumorigenesis, mitophagy helps maintain cellular homeostasis by removing damaged mitochondria, thereby preserving metabolic balance and preventing stress-induced cellular injury. This process contributes to tumor suppression (43). In the later stages of tumor development, cancer cells exploit mitophagy to adapt to hostile microenvironments, such as hypoxia and nutrient deprivation. By enhancing mitophagy, tumor cells increase their stress tolerance and survival capacity, ultimately promoting tumor progression (44).

Autophagy plays a dual role in the progression of digestive cancer. During the early transformation of normal cells into cancer cells, autophagy suppressed tumor growth and prevent cancer formation (45, 46). However, as tumors progress, autophagy inhibited apoptosis, enhanced neovascularization, and facilitated metastasis, driving continuous tumor cell proliferation (47). For example, as for the tumor-promoting function of autophagy. Huang et al. demonstrated that reduced Claudin5 expression enhanced malignant progression and radiotherapy resistance in esophageal squamous cell carcinoma via Beclin1-mediated autophagy (48). Wu et al. found that LncRNA SNHG11 drived gastric cancer progression by activating the Wnt/β-catenin pathway and promoting oncogenic autophagy (49). Qian et al. reported that thioredoxin related transmembrane protein 2 (TMX2) enhanced hepatocellular carcinoma cell viability by inducing autophagy and mitophagy (50). Devenport et al. revealed that colorectal cancer cells relied on autophagy to sustain mitochondrial metabolism to promoting proliferation under nutritional stress (51). Cui et al. discovered that fructose-induced mTORC1 activation accelerated pancreatic cancer progression by suppressing autophagy (52). Regarding the tumor-suppressive role of autophagy. Hong et al. found that Echinatin inhibited esophageal cancer growth and invasion by inducing AKT/mTOR-dependent autophagy and apoptosis (53). Wang et al. reported that knocking down PTBP1 (polypyrimidine tract-binding protein 1) suppressed gastric cancer progression by inducing oxidative stress via TXNIP (thioredoxin-interacting protein), disrupting autophagic flux (54). Zhang et al. found that DDX5 (DEAD box protein 5) inhibited liver tumorigenesis by promoting autophagy through interaction with p62/SQSTM1 (55). Huang et al. demonstrated that artesunate inhibited colorectal cancer cell growth by promoting ROS-dependent autophagy (56). Li et al. found that Sirtuin 4 activated autophagy by upregulating the p53 signaling pathway, thereby suppressing pancreatic cancer initiation and progression (57).

These findings underscore the close association between autophagy and digestive cancer progression. Thus, regulating autophagy expression is crucial for treating digestive cancers. Autophagy is regulated by various factors, including proteins, genes, and signaling pathways. For instance, mTOR, a key regulator of cell growth and metabolism, is a central pathway in autophagy regulation (58). Additionally, cancer-related genes (e.g., p53, p62, p21) and signaling pathways (e.g., Wnt/β-catenin, PI3K/AKT, AKT/mTOR) also regulate autophagy by either promoting or inhibiting its activity (59, 60).

## 3 Regulation of autophagy by circRNAs in digestive cancer

**Figure 3:**
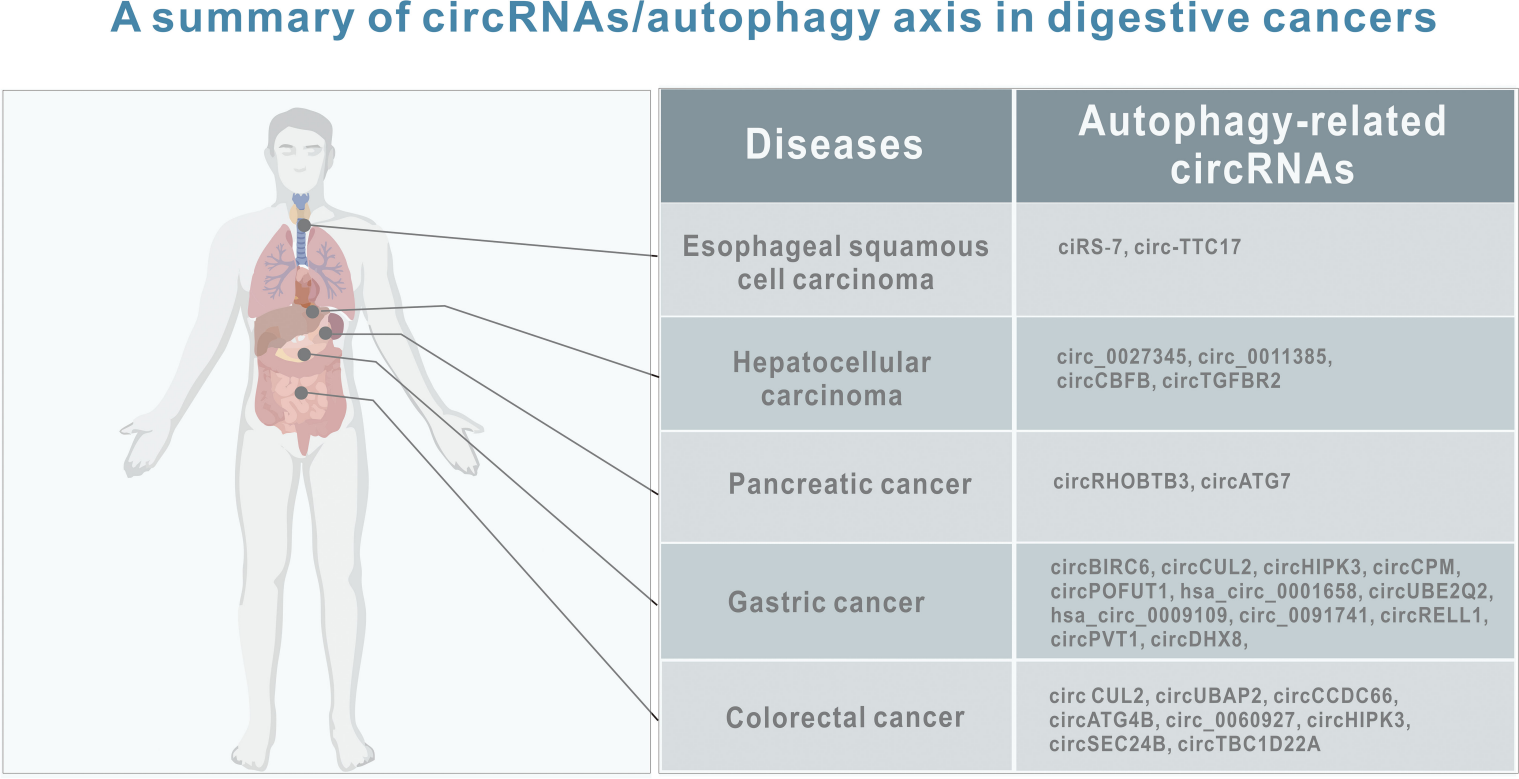
A summary of circRNAs/autophagy axis in digestive cancers. Tumors include esophageal squamous cell carcinoma, hepatocellular carcinoma, pancreatic cancer, gastric cancer and liver cancer. CircRNAs include ciRS‐7, circ-TTC17, circ_0027345, circ_0011385, circCBFB, circTGFBR2, circRHOBTB3, circATG7, circBIRC6, circCUL2, circHIPK3, circCPM, circPOFUT1, hsa_circ_0001658, circUBE2Q2, hsa_circ_0009109, circ_0091741, circRELL1,circPVT1, circDHX8, circ CUL2, circUBAP2, circCCDC66, circATG4B, circ_0060927, circHIPK3, circSEC24B, and circTBC1D22A.

**Figure 4:**
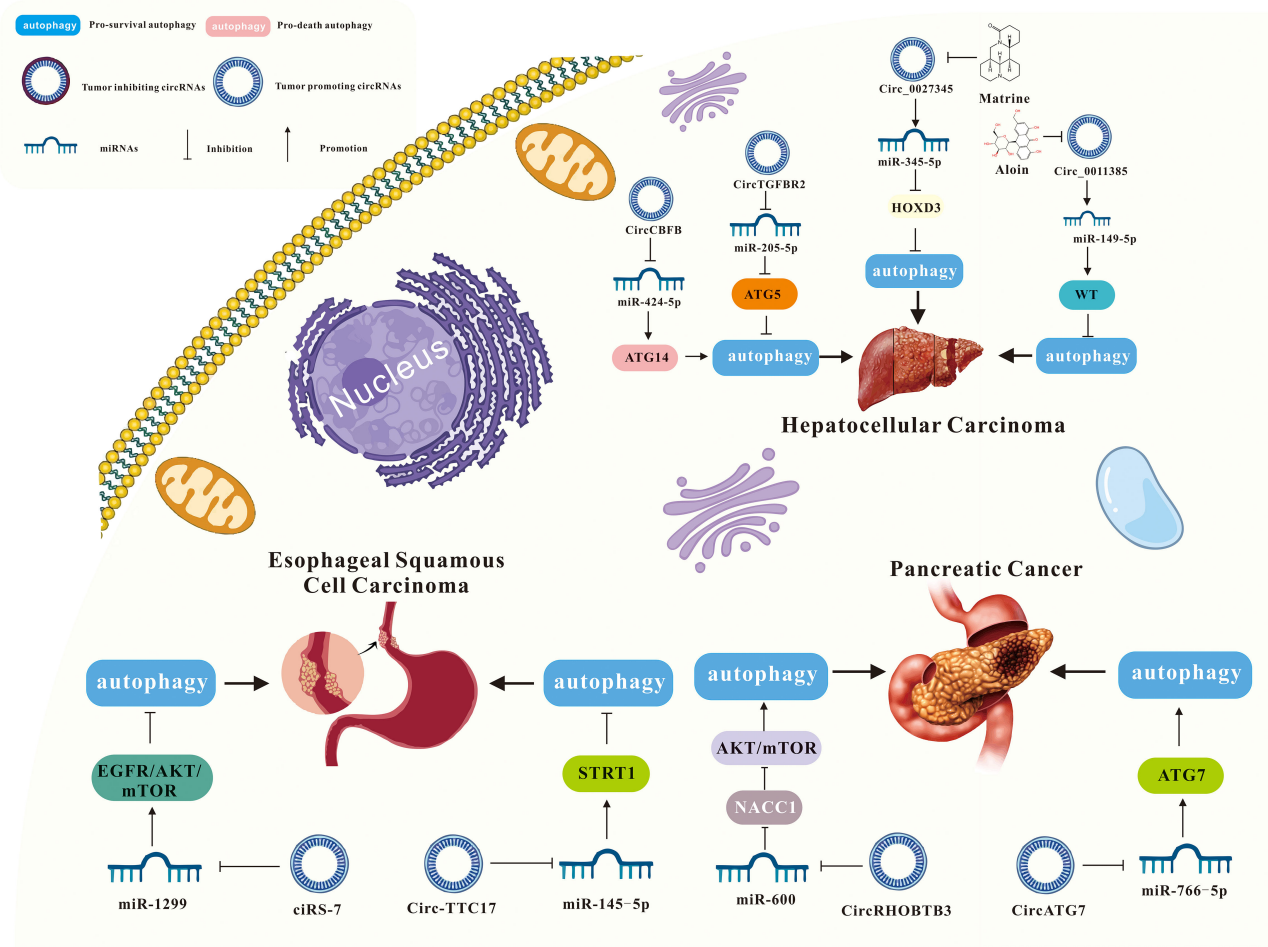
Interaction of circRNA and autophagy in ESCC, HCC and PC.

**Figure 5:**
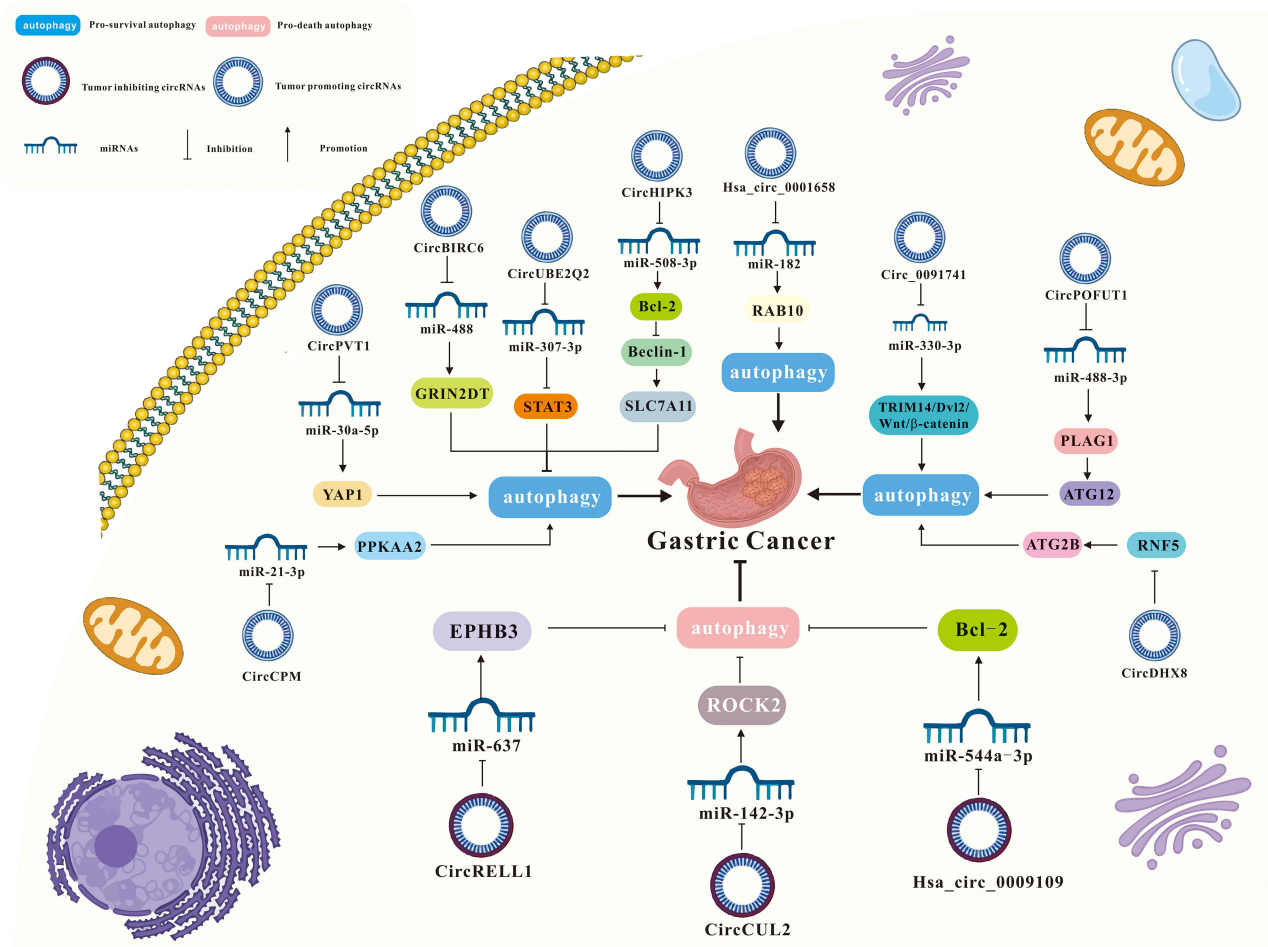
Interaction of circRNA and autophagy in GC.

**Figure 6:**
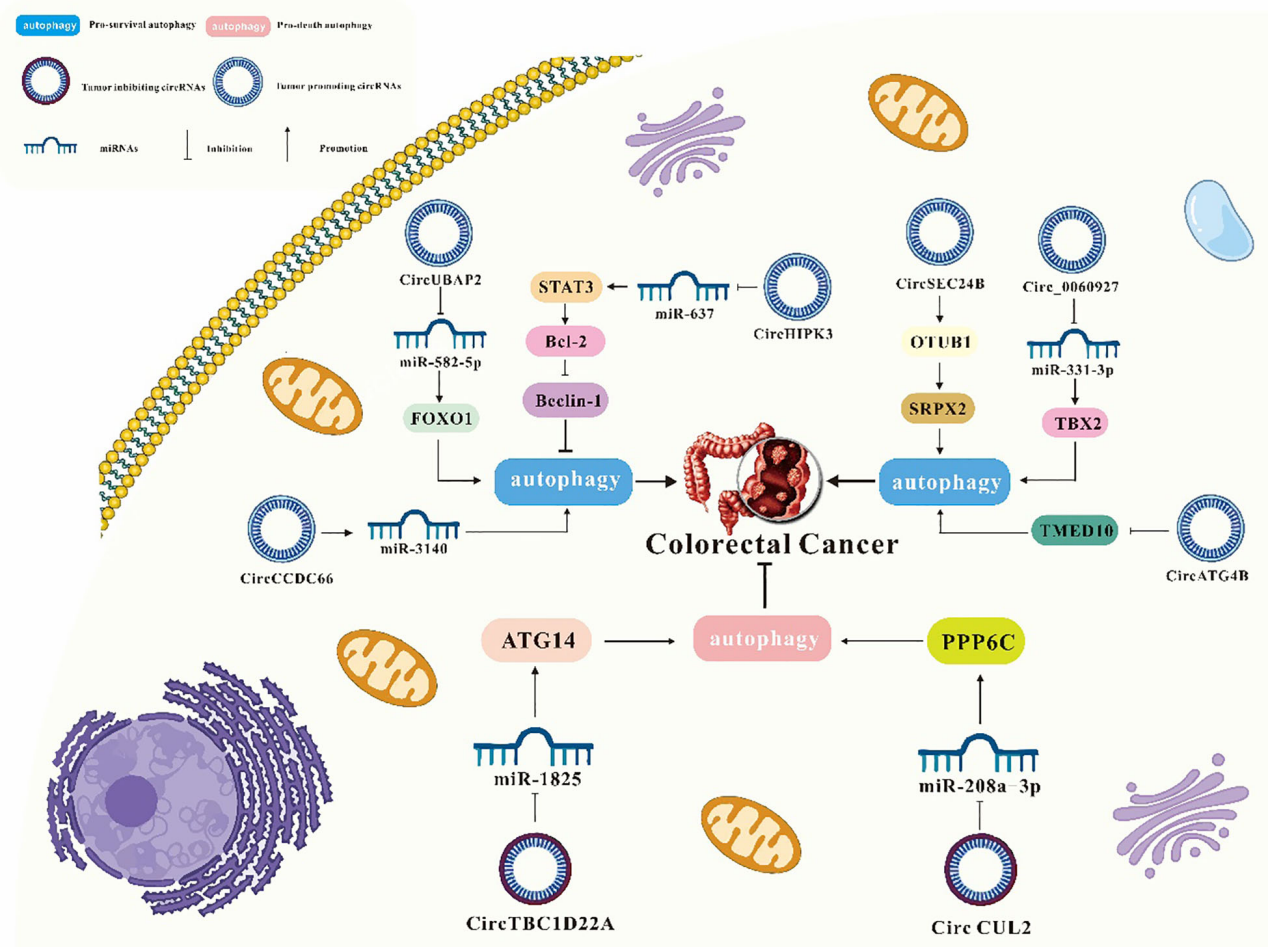
Interaction of circRNA and autophagy in CRC.

As mentioned above, more and more evidence suggests that circRNA-mediated autophagy plays an important role in the digestive system (**Figure 3**). Therefore, the roles of circRNA-mediated autophagy in esophageal squamous cell carcinoma (**Table 1**), hepatocellular carcinoma (**Table 1**), pancreatic cancer (**Table 1**), gastric cancer (**Table 2**) and colorectal cancer (**Table 3**) were introduced in the following sections. The specific mechanism of circRNA-mediated autophagy in different digestive system tumors is shown in **Figures 4**–**6**.

**Table 1:** The role of autophagy modulated by circRNAs in ESCC, HCC and PC.

Cancers	CircRNA	Target gene/ Regulatory pathway	Pro/Antitumor	Anti-/Proautophagy	References
ESCC	ciRS‐7	miR‐1299/EGFR/Akt/mTOR signaling	Pro-tumor	Antiautophagy	(61)
	circ-TTC17	miR‐145-5p/SIRT1 axis	Pro-tumor	Antiautophagy	(62)
HCC	circ_0027345	miR-345-5p/HOXD3 axis	Pro-tumor	Antiautophagy	(63)
	circ_0011385	miR-149-5p/WT1 axis	Pro-tumor	Antiautophagy	(64)
	circCBFB	miR-424-5p/ATG14 axis	Pro-tumor	Proautophagy	(65)
	circTGFBR2	miR-205-5p/ATG5 axis	Pro-tumor	Proautophagy	(66)
PC	circRHOBTB3	miR-600/NACC1/Akt/mTOR axis	Pro-tumor	Proautophagy	(67)
	circATG7	miR-766-5p/ATG7 axis	Pro-tumor	Proautophagy	(68)

**Table 2:** The role of autophagy modulated by circRNAs in GC.

CircRNA	Target gene/ Regulatory pathway	Pro/Antitumor	Anti-/Proautophagy	References
BIRC6	miR-488/GRIN2D-mediated CAV1-autophagy	Pro-tumor	Antiautophagy	(69)
CUL2	miR-142-3p/ROCK2 signaling	Antitumor	Antiautophagy	(70)
HIPK3	miR‐508‐3p/Bcl‐2/beclin1/SLC7A11 axis	Pro-tumor	Antiautophagy	(71)
CPM	miR-21-3p/PRKAA2 axis	Pro-tumor	Proautophagy	(72)
POFUT1	miR 488-3p/PLAG1-ATG12 axis	Pro-tumor	Proautophagy	(73)
hsa_circ_0001658	miR-182/RAB 10 axis	Pro-tumor	Proautophagy	(37)
UBE2Q2	miR-370-3p/STAT3 axis	Pro-tumor	Antiautophagy	(74)
hsa_circ_0009109	miR-544a-3p/Bcl-2 axis	Antitumor	Antiautophagy	(75)
circ_0091741	miR-330-3p/TRIM14/Dvl2/Wnt/β-catenin axis	Pro-tumor	Proautophagy	(76)
RELL1	miR-637/EPHB3 axis	Antitumor	Antiautophagy	(77)
PVT1	miR-30a-5p/YAP1 axis	Pro-tumor	Proautophagy	(78)
DHX8	RNF5/ATG2B axis	Pro-tumor	Proautophagy	(79)

**Table 3:** The role of autophagy modulated by circRNAs in CRC.

CircRNA	Target gene/ Regulatory pathway	Pro/Antitumor	Anti-/Proautophagy	References
circ CUL2	miR-208a-3p/PPP6C axis	Antitumor	Proautophagy	(80)
circUBAP2	miR-582-5p/FOXO1 signaling	Pro-tumor	Proautophagy	(81)
circCCDC66	miR-3140/autophagy pathway	Pro-tumor	Proautophagy	(82)
circATG4B	TMED10 signaling	Pro-tumor	Proautophagy	(83)
circ_0060927	miR-331-3p/TBX2 axis	Pro-tumor	Proautophagy	(84)
circHIPK3	miR-637/STAT3/Bcl-2/beclin1 axis	Pro-tumor	Antiautophagy	(85)
circSEC24B	OTUB1/SRPX2 axis	Pro-tumor	Proautophagy	(86)
circTBC1D22A	miR-1825/ATG14 axis	Antitumor	Proautophagy	(87)

### 3.1 Interaction of circRNAs and autophagy in esophageal squamous cell carcinoma

#### 3.1.1 ciRS‐7/miR‐1299/EGFR/Akt/mTOR signaling

Circular RNA ciRS-7 is dysregulated in the pathogenesis of several tumors, including gastric, colorectal, renal cell carcinoma, and pancreatic cancer (88–91). CiRS-7 functions as a molecular sponge, binding to specific miRNAs to regulate downstream gene expression and signaling pathways. Meng et al. reported that ciRS-7 inhibited autophagy in esophageal squamous cell carcinoma (ESCC) cells by acting as a miR-1299 sponge to target EGFR (epidermal growth factor receptor) signaling (61). Specifically, ciRS-7 suppressed starvation- or rapamycin-induced autophagy in ESCC cells. Conversely, miR-1299 promoted starvation or rapamycin-induced autophagy in ESCC cells by directly binding to the EGFR and influencing the downstream Akt-mTOR pathway. Therefore, ciRS-7, as a miR-1299 sponge, inhibited autophagy in ESCC cells by targeting EGFR signaling pathway (61).

#### 3.1.2 circ-TTC17/miR‐145-5p/SIRT1 axis

It has been reported that upregulated circ-TTC17 has been shown to promote proliferation and migration in ESCC cells. Wang et al. suggested that circ-TTC17 regulated ESCC progression by acting as a sponge for miRNAs (92). In the report conducted by Liu et al., it was observed that circ-TTC17 was overexpressed in ESCC, while miR-145-5p was downregulated. Silencing circ-TTC17 suppressed ESCC cell proliferation and metastasis, while enhancing apoptosis and autophagy-mediated radiosensitivity. Further research indicated that miR-145-5p inhibits ESCC progression. Additionally, silent information regulator sirtuin 1 (SIRT1), a downstream target of miR-145-5p, counteracts the inhibitory effects of miR-145-5p on ESCC progression when overexpressed. Therefore, circ-TTC17 promoted ESCC cell growth and metastasis via the miR-145-5p/SIRT1 axis while reducing autophagy-mediated radiosensitivity (62).

### 3.2 Interaction of circRNAs and autophagy in hepatocellular carcinoma

#### 3.2.1 circ_0027345/miR-345-5p/HOXD3 axis

Several studies have shown that circRNA plays a crucial role in tumor progression and gene regulation. In Sun et al.’s study, circRNA microarray analysis revealed that circ_0027345 was upregulated in hepatocellular carcinoma (HCC) tissues, and the results were confirmed by qRT-PCR (93). MiR-345-5p has been identified as an anti-cancer factor in several human cancers, including pancreatic cancer (94), gastric cancer (95) and lung cancer (96). The expression of miR-345 was downregulated in HCC tissues and cells, and its overexpression inhibited cell metastasis (97). Matrine, an alkaloid isolated from *Sophora flavescens* Ait, was associated with the proliferation, invasion, and metastasis of various tumors, including breast, liver, lung, and colorectal cancers (98). In an academic study conducted by Lin et al., Matrine inhibited the growth, migration, and invasion of HCC cells while promoting autophagy by upregulating miR-345-5p and downregulating circ_0027345 and homeobox D3 (HOXD3) (63).

#### 3.2.2 circ_0011385/miR-149-5p/WT1 axis

Circ_0011385 has been reported as a potential tumor progressor. Downregulation of circ_0011385 significantly inhibited cell proliferation and tumor activity in HCC (99). The expression of circ_0011385 could be inhibited by Aloin, and its overexpression reduced the anti-tumor effects of Aloin on HCC, which could be reversed by miR-149-5p. Furthermore, miR-149-5p regulated HCC progression by modulating WT1 expression. Therefore, Aloin inhibited cell proliferation, invasion, and tumor growth by regulating the circ_0011385/miR-149-5p/WT1 axis, and promoted HCC cell apoptosis and autophagy (64).

#### 3.2.3 circCBFB/miR-424-5p/ATG14 axis

CircCBFB has been shown to regulate cell proliferation, the cell cycle, and apoptosis via a competitive endogenous RNA (ceRNA) network in diseases like chronic lymphocytic leukemia and abdominal aortic aneurysm (100, 101). CircRNAs are known to promote cancer development by interacting with miRNAs in HCC (102). MiR-424-5p, located on human chromosome Xq26.3, functions as a tumor suppressor in liver cancer. Zhao et al. found that circCBFB and ATG14 (autophagy-related 14) were overexpressed, while miR-424-5p was downregulated in HCC tissues. Furthermore, overexpression of circCBFB or ATG14 enhanced HCC cell proliferation and autophagy, while suppressing apoptosis. Therefore, circCBFB suppressed miR-424-5p expression and upregulated ATG14, promoting the proliferation and autophagy of HCC cells (65).

#### 3.2.4 circTGFBR2/miR-205-5p/ATG5 axis

Transforming growth factor beta receptor II (TGFBR2), a transmembrane serine-threonine kinase, functions as a tumor suppressor gene, often downregulated in various cancers (103). Wang et al. showed that circTGFBR2 was delivered into HCC cells through exosomes as a competitive endogenous RNA, binding and promoting the degradation of miR-205-5p. This increased the expression of autophagy-related protein 5 (ATG5), thereby enhancing protective autophagy and facilitating HCC progression (66).

### 3.3 Interaction of circRNAs and autophagy in pancreatic cancer

#### 3.3.1 circRHOBTB3/miR-600/NACC1/Akt/mTOR axis

CircRHOBTB3 is generated through splicing and cyclization of the RHOBTB3 gene, which encodes a Rho GTPase family ATPase involved in membrane transport and proteasome degradation (104–106). Previous studies have identified circRHOBTB3 as a tumor suppressor circRNA in HCC (107). However, circRHOBTB3 is overexpressed in pancreatic ductal adenocarcinoma (PDAC). Yang et al. showed that circRHOBTB3 directly bound to miR-600, acting as a sponge to maintain the expression of its target gene, NACC1. This interaction promoted autophagy in PDAC cells via the Akt/mTOR pathway, supporting cell proliferation. Additionally, the RNA-binding protein FUS binds to pre-RHOBTB3 mRNA, facilitating the biosynthesis of circRHOBTB3. Therefore, circRHOBTB3 acted as a tumor activator, promoting PDAC cell proliferation through autophagy regulation via the miR-600/NACC1/Akt/mTOR axis (67).

#### 3.3.2 circATG7/miR-766-5p/ATG7 axis

Autophagy-related 7 (ATG7) is a key regulator of autophagy. It has been reported that ATG7 promoted pancreatic cancer (PC) progression by recruiting ATG5–12 complexes, which are highly expressed in PC tissues (108, 109). In an academic study conducted by He et al., circATG7 was significantly overexpressed in pancreatic cancer tissues and cell lines compared to adjacent normal tissues and pancreatic epithelial cells. Patients with high circATG7 expression had a poorer overall survival rate. CircATG7 also acted as a sponge for miR-766-5p, decreasing its expression and increasing the expression of its target gene, ATG7. Consequently, circATG7 accelerated PC progression through the miR-766-5p/ATG7 axis (68).

### 3.4 Interaction of circRNAs and autophagy in gastric cancer

#### 3.4.1 circ-BIRC6/miR-488/GRIN2D-mediated CAV1-autophagy signaling

In the autophagy and circRNA literature, the most widely studied digestive cancer is the gastric cancer. Previous studies have been identified BIRC6 as a high-risk gene present in various cancers (110–112). Yang et al. found that circBIRC6 promoted non-small cell lung cancer cell progression by sponging miRNA-145 (113). Recently in the report of Liu et al., it was demonstrated that circBIRC6 acts as a molecular sponge for miR-488, upregulating GRIN2D expression and promoting gastric cancer (GC) cell proliferation, migration, and invasion. Further studies have found that Overexpression of circBIRC6 increases GRIN2D expression, subsequently enhancing membrane caveolin-1 (CAV1) levels and inducing autophagy defects, significantly impacting tumorigenesis *in vivo*. Therefore, the circBIRC6/miR-488/GRIN2D axis promoted CAV1 expression in gastric cancer cells, leading to reduced autophagy (69).

#### 3.4.2 circCUL2/miR-142-3p/ROCK2 signaling

CircCUL2 is generated from the reverse splicing of CUL2 mRNA (from exon 2 to exon 4), located on chromosome 10:35,349,801-35,360,267, with a length of 339 nucleotides (nt). CircCUL2 is strongly associated with gastric cancer diagnosis, prognosis, tumor size, lymph node metastasis, tumor-lymph node-metastasis (TNM) staging, and treatment response (114). Moreover, research has demonstrated a strong association between circCUL2 and the advancement of colorectal cancer and non-small cell lung cancer (80, 115). Additionally, abnormal miR-142-3p expression correlates with gastric cancer stage (116). In an academic study conducted by Peng et al., it was found that circCUL2 was downregulated in gastric cancer tissues and cells. Its overexpression suppressed malignant transformation *in vitro* and tumorigenesis *in vivo*. Further studies have found that circCUL2 enhanced cisplatin sensitivity by sponging miR-142-3p to modulate ROCK2-mediated autophagy, thereby influencing tumor progression (70).

#### 3.4.3 circHIPK3/miR‐508‐3p/Bcl‐2/beclin1/SLC7A11 axis

CircHIPK3 has been reported to be dysregulated in various tumors. CircHIPK3 contributes to cancer progression through mechanisms like miRNA regulation and activation of traditional signaling pathways. Its effects include regulating the cell cycle, promoting cell proliferation, controlling apoptosis, and facilitating invasion and migration of cancer cells (117). Shang et al. found that circHIPK3 regulated cisplatin resistance in gastric cancer cells by modulating autophagy and ferroptosis via miR-508-3p/Bcl-2/beclin1/SLC7A11 axis (71).

#### 3.4.4 circCPM/miR-21-3p/PRKAA2 axis

CircCPM is derived from the fourth, fifth and sixth exons of the carboxypeptidase M (CPM) gene. The role of circCPM in tumors has not been previously reported. Recently, Recently, Fang et al. discovered that circCPM was upregulated in 5-FU-resistant gastric cancer cell lines and tissues, with high expression correlating with poor survival outcomes in gastric cancer. Additionally, silencing circCPM significantly enhanced chemosensitivity both *in vitro* and *in vivo*. Therefore, circCPM bound directly to miR-21-3p in the cytoplasm, increasing PRKAA2 expression, promoting autophagy activation, and contributing to chemotherapy resistance (72).

#### 3.4.5 circPOFUT1/miR-488-3p/PLAG1-ATG12 axis

Protein O-fucosyltransferase 1 (POFUT1) is an enzyme that adds serine or threonine residues to proteins through O-fucosylation and EGFR sequences via O-glycosidic bonds (118). POFUT1 has an oncogenic role in several cancers (119, 120). Dong et al. found that POFUT1 activated Notch/Wnt signaling pathways to promote gastric cancer development by the parafibromin-NICD1-β-catenin complex (121). Luo et al. showed that circPOFUT1 expression was upregulated in gastric cancer tissues and cells, and its increased levels are associated with poor prognosis. Further studies revealed that overexpression of circPOFUT1 enhanced the proliferation, migration, invasion and autophagy-related chemotherapy resistance of gastric cancer cells, while overexpression of miR-488-3p inhibited these effects. In addition, circPOFUT1 directly bound to miR-488-3p, thereby activating PLAG1 and ATG12 expression. Thus, circPOFUT1 promoted the proliferation, migration, invasion, and autophagy-related chemotherapy resistance of gastric cancer by activating PLAG1 and ATG12 through miR-488-3p binding (73).

#### 3.4.6 hsa_circ_0001658/miR-182/RAB 10 axis

Recent studies have identified circRNA_0001658 as a key biomarker for diagnosis and prognosis in tumor progression (122, 123). Duan et al. reported that circ_0001658 and RAB10 were upregulated, while miR-182 was downregulated in gastric cancer. Knockdown of circ_0001658 inhibited cell viability and autophagy in gastric cancer cells while increasing apoptosis. This inhibition was reversed upon downregulation of miR-182. Moreover, upregulation of RAB10 counteracted the effects of miR-182 on cell viability, autophagy, and apoptosis in gastric cancer cells. Therefore, silencing circ_0001658 inhibits RAB10 expression by sponging miR-182, thereby reducing cell viability, inhibiting autophagy, and promoting apoptosis in gastric cancer cells (37).

#### 3.4.7 circUBE2Q2/miR-370-3p/STAT3 axis

UBE2Q2, also known as Ubci, is a ubiquitin-conjugating enzyme that is often overexpressed in cancer. Signal transducer and activator of transcription 3 (STAT3) is an oncogene in the STAT protein family (124). Increasing evidence shows that abnormal STAT3 activation contributed to tumorigenesis by regulating autophagy, the cell cycle, glycolysis, and metastasis (125, 126). Additionally, STAT3 activation is linked to advanced cancer stages and metastasis (127, 128). Yang et al. found that CircUBE2Q2 is highly expressed in gastric cancer tissues and cell lines. Knockdown of circUBE2Q2 inhibited proliferation, migration, invasion, and glycolysis, while increasing autophagy *in vitro*. Additionally, circUBE2Q2 knockdown reduced tumorigenicity and metastasis *in vivo*. Therefore, circUBE2Q2 regulated gastric cancer progression via the circUBE2Q2/miR-370-3p/STAT3 axis and promoted metastasis through exosomal communication (74).

#### 3.4.8 hsa_circ_0009109/miR-544a-3p/Bcl-2 axis

Hsa_circ_0009109 has been shown to promote gastric cancer progression by accelerating cell proliferation, enhancing migration and invasion, inhibiting apoptosis, and speeding up cell cycle progression. Additionally, hsa_circ_0009109 reduced the expression of autophagy-related proteins, and an increase in autophagosomes was observed following interference with hsa_circ_0009109. Finally, hsa_circ_0009109 promoted tumor growth and induces autophagy by targeting the miR-544a-3p/Bcl-2 axis (75).

#### 3.4.9 circ_0091741/miR-330-3p/TRIM14/Dvl2/Wnt/β-catenin axis

Tripartite motif containing 14 (TRIM14), a member of the TRIM protein family, plays a role in several biological processes and is often dysregulated in human cancers (129). Wang et al. reported that TRIM14 regulated the AKT signaling pathway by activating miR-195-5p, which modulated epithelial-mesenchymal transition and promotes migration and invasion in gastric cancer (129). Chen et al. found that reduced expression of hsa_circ_0009109 promoted gastric cancer progression by accelerating cell proliferation, enhancing migration and invasion, inhibiting apoptosis, and accelerating the cell cycle. Moreover, hsa_circ_0009109 decreased autophagy-related protein expression, and interference with hsa_circ_0009109 resulted in an increase in autophagosomes. Finally, hsa_circ_0009109 promoted tumor growth and induces autophagy by targeting the miR-544a-3p/Bcl-2 axis (76).

#### 3.4.10 circRELL1/miR-637/EPHB3 axis

EPH receptor B3 (EPHB3) protein is a receptor tyrosine kinase that binds to transmembrane liver ligand B to initiate bidirectional signal transduction. Recently, it was reported that EphB3 gene is a tumor suppressor gene (130). Sang et al. reported that circRELL1 block cell proliferation, invasion, migration and anti-apoptosis in patients with gastric cancer. Further studies have shown that circRELL1 adsorbed miR-637 and indirectly up-regulate the expression of EPHB3 by regulating the activation of autophagy in GC. In addition, circRELL1 inhibited the malignant behavior of gastric cancer *in vitro* and *in vivo* through the exosome pathway. Therefore, exosomal circRELL1 regulated gastric cancer progression by regulating autophagy activation through the miR-637/EPHB3 axis (77).

#### 3.4.11 circ-PVT1/miR-30a-5p/YAP1 axis

Circ-PVT1, derived from exon 2 of the PVT1 gene, forms a closed-loop structure through reverse splicing (131). As an oncogene, Circ-PVT1 is aberrantly expressed in various tumor types, including cervical, gastric, colorectal cancers, pancreatic ductal adenocarcinoma, and oral squamous cell carcinoma, and is associated with poor prognosis in these malignancies (132). Yao et al. reported that exosomal circ-PVT1 was upregulated in cisplatin (DDP)-resistant gastric cancer serum and cells, while miR-30a-5p expression was downregulated. Further studies revealed that knockdown of circ-PVT1 promoted apoptosis and reduced invasion and autophagy by negatively targeting miR-30a-5p, thus inhibiting cisplatin resistance in resistant gastric cancer cells. Additionally, Yes1 associated transcriptional regulator (YAP1) was identified as a direct target of miR-30a-5p. Overexpression of miR-30a-5p inhibited DDP resistance by downregulating YAP1. Therefore, exosomal circPVT1 regulates autophagy, invasion, and apoptosis in gastric cancer cells via the miR-30a-5p/YAP1 axis, promoting cisplatin resistance (78).

#### 3.4.12 circDHX8/RNF5/ATG2B axis

CircDHX8 (Hsa_circ_0003899) is a recently identified circular RNA that plays a significant role in gastric cancer. RNF5 (also known as RMA1) is an E3 ubiquitin ligase involved in various physiological processes, including protein localization and cancer progression. For instance, Zhang et al. demonstrated that prostaglandin D2 (PGD2) competed with ATG4B to bind to RNF5, promoting ATG4B expression and influencing the self-renewal capacity of gastric cancer stem cells (133). In the academic study by Wei et al., it was found that circDHX8 stabilizes ATG2B by binding to the ubiquitinating enzyme RNF5, preventing the interaction between ATG2B and RNF5. Furthermore, acetylated ATG2B undergoes SIRT1-mediated deacetylation, which enhances its binding to RNF5, thereby promoting autophagy and tumor progression in gastric cancer (79).

### 3.5 Interaction of circRNAs and autophagy in colorectal cancer

#### 3.5.1 circ CUL2/miR-208a-3p/PPP6C axis

CircCUL2 functions as a tumor suppressor in various cancers. In colorectal cancer (CRC), it has been shown to inhibit cell proliferation and promote apoptosis and autophagy. Yang et al. reported that circCUL2 was downregulated in CRC tissues and cell lines. Overexpression of circCUL2 suppressed CRC cell proliferation while inducing apoptosis and autophagy. CircCUL2 interacted with miR-208a-3p and regulates PPP6C expression. By sponging miR-208a-3p, circCUL2 inhibited CRC progression (80).

#### 3.5.2 circUBAP2/miR-582-5p/FOXO1 signaling

CircUBAP2, originating from the ubiquitin-associated protein 2 gene, forms a closed-loop structure via reverse splicing. It plays a crucial role in tumor cell growth and metastasis across various cancers (134). Chen et al. discovered that circUBAP2 was highly expressed in CRC tissues and cell lines, promoting autophagy both *in vitro* and *in vivo*. CircUBAP2 directly interacts with miR-582-5p, acting as a molecular sponge. This interaction modulates the expression of its target gene, *forkhead box protein O1* (*FOXO1*), and associated downstream signaling pathways. Consequently, circUBAP2 promoted CRC progression and metastasis via the circUBAP2/miR-582-5p/FOXO1 axis (81).

#### 3.5.3 circCCDC66/miR-3140/autophagy pathway

Recent studies have shown that circCCDC66 is overexpressed in colon cancer, contributing to tumor growth and metastasis (82, 135). Feng et al. found that hypoxia increases circCCDC66 expression, thereby facilitating CRC progression. Knockdown of circCCDC66 significantly decreased the viability, migration, and invasion of hypoxia-exposed CRC cells while promoting apoptosis. Furthermore, miR-3140 deletion partially reversed the effects of circCCDC66 on the phenotype of hypoxic CRC cells. Thus, circCCDC66 promoted CRC progression under hypoxic conditions by modulating the miR-3140/autophagy axis (136).

#### 3.5.4 circATG4B/TMED10 signaling

In an academic study conducted by Pan et al., ATG4B-derived circRNA (circATG4B) was increased in exosomes secreted by oxaliplatin-resistant CRC cells. Additional research revealed that circATG4B encoded a novel protein, circATG4B-222aa, which competitively bound to Transmembrane P24 trafficking protein 10 (TMED10). This interaction inhibited TMED10 binding to ATG4B, leading to enhanced autophagy and chemotherapy resistance (83).

#### 3.5.5 circ_0060927/miR-331-3p/TBX2 axis

Circ_0060927, originating from the CYP24A1 gene (located in chr20:52773707–52788209), is reported to be overexpressed in CRC (84, 137). Yin et al. demonstrated that deletion of circ_0060927 suppressed CRC cell proliferation, autophagy, migration, and invasion, while enhancing apoptosis and necrosis and reducing tumor growth *in vivo*. Circ_0060927 acted as a molecular sponge for miR-331-3p, thereby upregulating its downstream target gene, T-Box transcription factor 2 (TBX2), to drive cancer progression. Therefore, circ_0060927 facilitated CRC development by sponging miR-331-3p and upregulating TBX2 (84).

#### 3.5.6 circHIPK3/miR-637/STAT3/Bcl-2/beclin1 axis

CircRNA homologous domain interacting protein kinase 3 (CircHIPK3) is implicated in human cancers (138). Extensive research highlights circHIPK3 as a miRNA sponge that regulates target genes to influence cell proliferation, invasion, and migration (139). Zhang et al. reported that circHIPK3 expression was increased in chemotherapy-resistant CRC patients. CircHIPK3 was found to enhance oxaliplatin resistance in CRC by suppressing autophagy. CircHIPK3 sponged miR-637, upregulated STAT3 expression, and activates the downstream Bcl-2/beclin1 signaling pathway, contributing to oxaliplatin resistance in CRC (85).

#### 3.5.7 circSEC24B/OTUB1/SRPX2 axis

Wang et al. found that the expression of circSEC24B increased in CRC tissues and cell lines, and enhanced the proliferation and autophagy of CRC cells. Specifically, circSEC24B functioned as a scaffold to promote the binding of OTU Deubiquitinase, Ubiquitin Aldehyde Binding 1 (OTUB1) to Sushi repeat containing protein X-linked 2 (SRPX2), thereby enhancing its protein stability. In addition, OTUB1 regulated the expression of SRPX2 through an acetylation-dependent mechanism. In summary, circSEC24B activated autophagy by promoting the deubiquitination of SRPX2 and induced chemoresistance in CRC through the deubiquitinating enzyme OTUB1 (86).

#### 3.5.8 circTBC1D22A/miR-1825/ATG14 axis

Growing evidence suggests that circRNAs play a key role in cancer progression and metastasis. Sun et al. found that miR-1825 was highly expressed in CRC tissues and positively correlated with lymph node metastasis and distant metastasis. In addition, *in vitro* and *in vivo* experiments confirmed that miR-1825 was positively correlated with the proliferation and migration of CRC cells. CircTBC1D22A inhibited this event. Therefore, CircTBC1D22A directly interacted with miR-1825 and then acted as a miRNA sponge to regulate the expression of the target gene ATG14 and jointly promote autophagy-mediated CRC progression and metastasis (87).

## 4 Therapeutic potential related to circRNAs-autophagy mechanism

According to current evidence, circRNA-mediated autophagy regulation can affect the sensitivity of digestive system tumor cells to radiotherapy and chemotherapy (140). After radiotherapy or chemotherapy, tumor patients often trigger autophagy of tumor cells. Because autophagy is an important process for maintaining cellular energy balance and homeostasis, cancer cells may provide the energy needed to escape radiation and chemotherapy-induced apoptosis through autophagy, leading to treatment resistance (108). Studies have shown that autophagy-related circRNA mediates the resistance of tumor cells to radiotherapy and chemotherapy by affecting cell processes such as proliferation, apoptosis, migration, invasion, drug resistance, and angiogenesis in digestive system tumors (gastric cancer and colorectal cancer) (26, 38, 141). For instance, Fang et al. reported that circCPM promoted chemoresistance of gastric cancer by activating PRKAA2-mediated autophagy (72), while Luo et al. showed that circPOFUT1 promoted the proliferation, migration, invasion, and autophagy-related chemotherapy resistance of gastric cancer by activating PLAG1 and ATG12 through miR-488-3p binding (73). As for colorectal cancer, Wang et al. demonstrated that circSEC24B activated autophagy through OTUB1-mediated SRPX2 deubiquitination and induced chemoresistance in colorectal cancer (86). These findings provide new insights into the role of circRNA-mediated autophagy in digestive system radiotherapy and chemotherapy. Abnormally expressed circRNAs in digestive system tumors may work together with autophagy to determine the response of tumor cells to radiation and drug treatment, so this is a key area that needs further research.

## 5 Limitations and future perspectives

This study systematically reviewed the relationship between circRNAs and autophagy in digestive system tumors. The findings suggest that circRNAs in digestive system tumors influence cancer progression, including cell proliferation, apoptosis, migration, invasion, and angiogenesis. It highlights the dual role of circRNAs in either activating or inhibiting autophagy. Similarly, autophagy exerts a dual regulatory effect on digestive system cancer progression. For example, in gastric cancer, circRNAs such as CPM, POFUT1, and hsa_circ_0001658 promote cancer progression by activating autophagy, while CUL2 and UBE2Q2 inhibit progression via autophagy activation. Conversely, HIPK3 promotes gastric cancer progression by inhibiting autophagy, whereas hsa_circ_0009109 inhibits progression through autophagy suppression. Despite these insights, limitations remain in understanding the role of autophagy-related circRNAs in digestive system cancers. (1) Individual differences: the patient heterogeneity and tumor microenvironment complexity result in significant variability in circRNA expression and autophagy activity, complicating the assessment of their roles in tumor progression. (2) Unclear mechanisms: The dual role of autophagy in tumor progression and its intricate pathways make its exact mechanisms in digestive system cancers difficult to decipher. Moreover, whether circRNA-based therapies rely solely on autophagy remains uncertain and requires further study. (3) Limited clinical research: Current findings are largely based on preclinical models, with minimal clinical studies. Thus, the diagnostic, therapeutic, and prognostic potential of circRNAs and autophagy in digestive system cancers requires further investigation.

Future research should focus on the interplay between circRNA-mediated autophagy and other mechanisms driving tumor progression within the digestive system tumor microenvironment. Unraveling these mechanisms could pave the way for identifying valuable diagnostic and prognostic biomarkers, as well as developing innovative therapeutic strategies for digestive malignancies. This review highlights the significant role of circRNA-related autophagy in the progression of digestive system tumors, such as esophageal, gastric, pancreatic, liver, and colorectal cancers. CircRNA regulation can either promote or inhibit autophagy in these malignancies. The intricate network of circRNA-targeted genes and signaling pathways governs autophagy regulation, influencing the initiation and progression of digestive system tumors. Therefore, targeting these circRNAs to modulate autophagy represents a promising approach for developing reliable diagnostic and prognostic biomarkers, as well as effective therapeutic strategies for digestive system tumors.

## References

[1] Kuntz S Krieghoff-Henning E Kather JN Jutzi T Höhn J Kiehl L. Gastrointestinal cancer classification and prognostication from histology using deep learning: Systematic review. Eur J Cancer. (2021) 155:200–15. doi: 10.1016/j.ejca.2021.07.012, PMID: 34391053

[2] Danpanichkul P Suparan K Tothanarungroj P Dejvajara D Rakwong K Pang Y. Epidemiology of gastrointestinal cancers: a systematic analysis from the global burden of disease study 2021. Gut. (2025) 74:26–34. doi: 10.1136/gutjnl-2024-333227, PMID: 39242191

[3] Global cancer burden growing, amidst mounting need for services. Saudi Med J. (2024) 45:326–7., PMID: 38438207 PMC11115397

[4] Bray F Laversanne M Sung H Ferlay J Siegel RL Soerjomataram I. Global cancer statistics 2022: GLOBOCAN estimates of incidence and mortality worldwide for 36 cancers in 185 countries. CA Cancer J Clin. (2024) 74:229–63. doi: 10.3322/caac.21834, PMID: 38572751

[5] Arnold M Abnet CC Neale RE Vignat J Giovannucci EL McGlynn KA. Global burden of 5 major types of gastrointestinal cancer. Gastroenterology. (2020) 159:335–49.e15. doi: 10.1053/j.gastro.2020.02.068, PMID: 32247694 PMC8630546

[6] Chen S Cao Z Prettner K Kuhn M Yang J Jiao L. Estimates and projections of the global economic cost of 29 cancers in 204 countries and territories from 2020 to 2050. JAMA Oncol. (2023) 9:465–72. doi: 10.1001/jamaoncol.2022.7826, PMID: 36821107 PMC9951101

[7] Chen L Huang C Wang X Shan G. Circular RNAs in eukaryotic cells. Curr Genomics. (2015) 16:312–8. doi: 10.2174/1389202916666150707161554, PMID: 27047251 PMC4763969

[8] Salzman J. Circular RNA expression: its potential regulation and function. Trends Genet. (2016) 32:309–16. doi: 10.1016/j.tig.2016.03.002, PMID: 27050930 PMC4948998

[9] Han B Chao J Yao H. Circular RNA and its mechanisms in disease: from the bench to the clinic. Pharmacol Ther. (2018) 187:31–44. doi: 10.1016/j.pharmthera.2018.01.010, PMID: 29406246

[10] Conn VM Chinnaiyan AM Conn SJ. Circular RNA in cancer. Nat Rev Cancer. (2024) 24:597–613. doi: 10.1038/s41568-024-00721-7, PMID: 39075222

[11] Altesha MA Ni T Khan A Liu K Zheng X. Circular RNA in cardiovascular disease. J Cell Physiol. (2018) 234:5588–6000. doi: 10.1002/jcp.27384, PMID: 30341894

[12] Zhou X Ao X Jia Z Li Y Kuang S Du C. Non-coding RNA in cancer drug resistance: Underlying mechanisms and clinical applications. Front Oncol. (2022) 12:951864. doi: 10.3389/fonc.2022.951864, PMID: 36059609 PMC9428469

[13] Yun BD Choi YJ Son SW Cipolla GA Berti FCB Malheiros D. Oncogenic role of exosomal circular and long noncoding RNAs in gastrointestinal cancers. Int J Mol Sci. (2022) 23:930. doi: 10.3390/ijms23020930, PMID: 35055115 PMC8781283

[14] Li Z Ruan Y Zhang H Shen Y Li T Xiao B. Tumor-suppressive circular RNAs: mechanisms underlying their suppression of tumor occurrence and use as therapeutic targets. Cancer Sci. (2019) 110:3630–8. doi: 10.1111/cas.14211, PMID: 31599076 PMC6890437

[15] Du A Li S Zhou Y Disoma C Liao Y Zhang Y. M6A-mediated upregulation of circMDK promotes tumorigenesis and acts as a nanotherapeutic target in hepatocellular carcinoma. Mol Cancer. (2022) 21:109. doi: 10.1186/s12943-022-01575-z, PMID: 35524319 PMC9074191

[16] Wang Z Liu C. Upregulated hsa_circRNA_100269 inhibits the growth and metastasis of gastric cancer through inactivating PI3K/Akt axis. PloS One. (2021) 16:e0250603. doi: 10.1371/journal.pone.0250603, PMID: 33901239 PMC8075232

[17] Zada S Hwang JS Ahmed M Lai TH Pham TM Elashkar O. Cross talk between autophagy and oncogenic signaling pathways and implications for cancer therapy. Biochim Biophys Acta Rev Cancer. (2021) 1876:188565. doi: 10.1016/j.bbcan.2021.188565, PMID: 33992723

[18] Niu X You Q Hou K Tian Y Wei P Zhu Y. Autophagy in cancer development, immune evasion, and drug resistance. Drug Resist Updat. (2025) 78:101170. doi: 10.1016/j.drup.2024.101170, PMID: 39603146

[19] Shao BZ Zhang WG Liu ZY Linghu EQ. Autophagy and its role in gastrointestinal diseases. World J Gastroenterol. (2024) 30:4014–20. doi: 10.3748/wjg.v30.i36.4014, PMID: 39351250 PMC11439115

[20] Cao Q Bai P. Role of autophagy in renal cancer. J Cancer. (2019) 10:2501–9. doi: 10.7150/jca.29285, PMID: 31258756 PMC6584354

[21] Folkerts H Hilgendorf S Vellenga E Bremer E Wiersma VR. The multifaceted role of autophagy in cancer and the microenvironment. Med Res Rev. (2018) 39:517–60. doi: 10.1002/med.21531, PMID: 30302772 PMC6585651

[22] Denton D Kumar S. Autophagy-dependent cell death. Cell Death Differ. (2019) 26:605–16. doi: 10.1038/s41418-018-0252-y, PMID: 30568239 PMC6460387

[23] Saadh MJ Ehymayed HM Alazzawi TS Fahdil AA Athab ZH Yarmukhamedov B. Role of circRNAs in regulating cell death in cancer: a comprehensive review. Cell Biochem Biophys. (2025) 83:109–33. doi: 10.1007/s12013-024-01492-6, PMID: 39243349

[24] Liang G Ling Y Mehrpour M Saw PE Liu Z Tan W. Autophagy-associated circRNA circCDYL augments autophagy and promotes breast cancer progression. Mol Cancer. (2020) 19:65. doi: 10.1186/s12943-020-01152-2, PMID: 32213200 PMC7093993

[25] Gan X Zhu H Jiang X Obiegbusi SC Yong M Long X. CircMUC16 promotes autophagy of epithelial ovarian cancer via interaction with ATG13 and miR-199a. Mol Cancer. (2020) 19:45. doi: 10.1186/s12943-020-01163-z, PMID: 32111227 PMC7047414

[26] Wang Y Mo Y Peng M Zhang S Gong Z Yan Q. The influence of circular RNAs on autophagy and disease progression. Autophagy. (2021) 18:240–53. doi: 10.1080/15548627.2021.1917131, PMID: 33904341 PMC8942425

[27] Liu G Zhang Z Song Q Guo Y Bao P Shui H. Circ_0006528 contributes to paclitaxel resistance of breast cancer cells by regulating miR-1299/CDK8 axis. Onco Targets Ther. (2020) 13:9497–511. doi: 10.2147/OTT.S252886, PMID: 33061434 PMC7522311

[28] Zhu L Wang C Lin S Zong L. CircKIAA0907 retards cell growth, cell cycle, and autophagy of gastric cancer *in vitro* and inhibits tumorigenesis *in vivo* via the miR-452-5p/KAT6B axis. Med Sci Monit. (2020) 26:e924160. doi: 10.12659/MSM.924160, PMID: 32722658 PMC7412918

[29] Sun G Li Z He Z Wang W Wang S Zhang X. Circular RNA MCTP2 inhibits cisplatin resistance in gastric cancer by miR-99a-5p-mediated induction of MTMR3 expression. J Exp Clin Cancer Res. (2020) 39:246. doi: 10.1186/s13046-020-01758-w, PMID: 33198772 PMC7670601

[30] Wang T He M Zhang X Guo Z Wang P Long F. Deciphering the impact of circRNA-mediated autophagy on tumor therapeutic resistance: a novel perspective. Cell Mol Biol Lett. (2024) 29:60. doi: 10.1186/s11658-024-00571-z, PMID: 38671354 PMC11046940

[31] Xu Z Han X Ou D Liu T Li Z Jiang G. Targeting PI3K/AKT/mTOR-mediated autophagy for tumor therapy. Appl Microbiol Biotechnol. (2019) 104:575–87. doi: 10.1007/s00253-019-10257-8, PMID: 31832711

[32] Xue L Jia T Zhu Y Zhao L Mao J. Down-regulation of circ_0058058 suppresses proliferation, angiogenesis and metastasis in multiple myeloma through miR-338-3p/ATG14 pathway. J Orthop Surg Res. (2021) 16:723. doi: 10.1186/s13018-021-02867-8, PMID: 34930344 PMC8686392

[33] Cui Y Cao J Huang S Ye J Huang H Liao D. CircRNA_0006470 promotes the proliferation and migration of gastric cancer cells by functioning as a sponge of miR-27b-3p. Neoplasma. (2021) 68:1245–56. doi: 10.4149/neo_2021_210222N235, PMID: 34641696

[34] Zhong C Wu K Wang S Long Z Yang T Zhong W. Autophagy-related circRNA evaluation reveals hsa_circ_0001747 as a potential favorable prognostic factor for biochemical recurrence in patients with prostate cancer. Cell Death Dis. (2021) 12:726. doi: 10.1038/s41419-021-04015-w, PMID: 34294687 PMC8298711

[35] Wei H Li L Zhang H Xu F Chen L Che G. Circ-FOXM1 knockdown suppresses non-small cell lung cancer development by regulating the miR-149-5p/ATG5 axis. Cell Cycle. (2021) 20:166–78. doi: 10.1080/15384101.2020.1867780, PMID: 33413028 PMC7889128

[36] Liu H Yuan HF Xu D Chen KJ Tan N Zheng QJ. Circular RNA circ_0000034 upregulates STX17 level to promote human retinoblastoma development via inhibiting miR-361-3p. Eur Rev Med Pharmacol Sci. (2020) 24:12080–92. doi: 10.26355/eurrev_202012_23997, PMID: 33336726

[37] Duan X Yu X Li Z. Circular RNA hsa_circ_0001658 regulates apoptosis and autophagy in gastric cancer through microRNA-182/Ras-related protein Rab-10 signaling axis. Bioengineered. (2022) 13:2387–97. doi: 10.1080/21655979.2021.2024637, PMID: 35030981 PMC8974080

[38] Zhou Z Zhang Y Gao J Hao X Shan C Li J. Circular RNAs act as regulators of autophagy in cancer. Mol Ther Oncolytics. (2021) 21:242–54. doi: 10.1016/j.omto.2021.04.007, PMID: 34095462 PMC8142048

[39] Mizushima N Komatsu M. Autophagy: renovation of cells and tissues. Cell. (2011) 147:728–41. doi: 10.1016/j.cell.2011.10.026, PMID: 22078875

[40] Mizushima N Levine B Cuervo AM Klionsky DJ. Autophagy fights disease through cellular self-digestion. Nature. (2008) 451:1069–75. doi: 10.1038/nature06639, PMID: 18305538 PMC2670399

[41] Um JH Yun J. Emerging role of mitophagy in human diseases and physiology. BMB Rep. (2017) 50:299–307. doi: 10.5483/bmbrep.2017.50.6.056, PMID: 28366191 PMC5498140

[42] Soengas MS. Mitophagy or how to control the Jekyll and Hyde embedded in mitochondrial metabolism: implications for melanoma progression and drug resistance. Pigment Cell Melanoma Res. (2012) 25:721–31. doi: 10.1111/pcmr.12021, PMID: 22974269

[43] Qiu YH Zhang TS Wang XW Wang MY Zhao WX Zhou HM. Mitochondria autophagy: a potential target for cancer therapy. J Drug Targeting. (2021) 29:576–91. doi: 10.1080/1061186X.2020.1867992, PMID: 33554661

[44] Mazure NM Brahimi-Horn MC Pouysségur J. Hypoxic mitochondria: accomplices in resistance. Bull Cancer. (2011) 98:40–6. doi: 10.1684/bdc.2011.1360, PMID: 21609892

[45] Akkoç Y Gözüaçık D. Autophagy and liver cancer. Turk J Gastroenterol. (2018) 29:270–82. doi: 10.5152/tjg.2018.150318, PMID: 29755011 PMC6284658

[46] Chao X Qian H Wang S Fulte S Ding WX. Autophagy and liver cancer. Clin Mol Hepatol. (2020) 26:606–17. doi: 10.3350/cmh.2020.0169, PMID: 33053934 PMC7641568

[47] Wen W Ertas YN Erdem A Zhang Y. Dysregulation of autophagy in gastric carcinoma: Pathways to tumor progression and resistance to therapy. Cancer Lett. (2024) 591:216857. doi: 10.1016/j.canlet.2024.216857, PMID: 38583648

[48] Huang S Zhang J Li Y Xu Y Jia H An L. Downregulation of Claudin5 promotes Malignant progression and radioresistance through Beclin1-mediated autophagy in esophageal squamous cell carcinoma. J Transl Med. (2023) 21:379. doi: 10.1186/s12967-023-04248-7, PMID: 37303041 PMC10257837

[49] Wu Q Ma J Wei J Meng W Wang Y Shi M. lncRNA SNHG11 promotes gastric cancer progression by activating the Wnt/β-catenin pathway and oncogenic autophagy. Mol Ther. (2021) 29:1258–78. doi: 10.1016/j.ymthe.2020.10.011, PMID: 33068778 PMC7934455

[50] Zhang W Tang Y Yang P Chen Y Xu Z Qi C. TMX2 potentiates cell viability of hepatocellular carcinoma by promoting autophagy and mitophagy. Autophagy. (2024) 20:2146–63. doi: 10.1080/15548627.2024.2358732, PMID: 38797513 PMC11423672

[51] Devenport SN Singhal R Radyk MD Taranto JG Kerk SA Chen B. Colorectal cancer cells utilize autophagy to maintain mitochondrial metabolism for cell proliferation under nutrient stress. JCI Insight. (2021) 6:e138835. doi: 10.1172/jci.insight.138835, PMID: 34138755 PMC8328084

[52] Cui Y Tian J Wang Z Guo H Zhang H Wang Z. Fructose-induced mTORC1 activation promotes pancreatic cancer progression through inhibition of autophagy. Cancer Res. (2023) 83:4063–79. doi: 10.1158/0008-5472.CAN-23-0464, PMID: 37738413 PMC10722142

[53] Hong P Liu QW Xie Y Zhang QH Liao L He QY. EChinatin suppresses esophageal cancer tumor growth and invasion through inducing AKT/mTOR-dependent autophagy and apoptosis. Cell Death Dis. (2020) 11:524. doi: 10.1038/s41419-020-2730-7, PMID: 32655130 PMC7354992

[54] Wang S Wang X Qin C Liang C Li W Ran A. PTBP1 knockdown impairs autophagy flux and inhibits gastric cancer progression through TXNIP-mediated oxidative stress. Cell Mol Biol Lett. (2024) 29:110. doi: 10.1186/s11658-024-00626-1, PMID: 39153986 PMC11330137

[55] Zhang H Zhang Y Zhu X Chen C Zhang C Xia Y. DEAD box protein 5 inhibits liver tumorigenesis by stimulating autophagy via interaction with p62/SQSTM1. Hepatology. (2019) 69:1046–63. doi: 10.1002/hep.30300, PMID: 30281815 PMC6411283

[56] Huang Z Gan S Zhuang X Chen Y Lu L Wang Y. Artesunate inhibits the cell growth in colorectal cancer by promoting ROS-dependent cell senescence and autophagy. Cells. (2022) 11:2472. doi: 10.3390/cells11162472, PMID: 36010550 PMC9406496

[57] Li J Zhan H Ren Y Feng M Wang Q Jiao Q. Sirtuin 4 activates autophagy and inhibits tumorigenesis by upregulating the p53 signaling pathway. Cell Death Differ. (2022) 30:313–26. doi: 10.1038/s41418-022-01063-3, PMID: 36209169 PMC9950374

[58] Mo Y Sun YY Liu KY. Autophagy and inflammation in ischemic stroke. Neural Regener Res. (2020) 15:1388–96. doi: 10.4103/1673-5374.274331, PMID: 31997797 PMC7059569

[59] Shen M Li X Qian B Wang Q Lin S Wu W. Crucial roles of microRNA-mediated autophagy in urologic Malignancies. Int J Biol Sci. (2021) 17:3356–68. doi: 10.7150/ijbs.61175, PMID: 34512152 PMC8416737

[60] Gan L Zheng L Zou J Luo P Chen T Zou J. Critical roles of lncRNA-mediated autophagy in urologic Malignancies. Front Pharmacol. (2024) 15:1405199. doi: 10.3389/fphar.2024.1405199, PMID: 38939836 PMC11208713

[61] Meng L Liu S Ding P Chang S Sang M. Circular RNA ciRS-7 inhibits autophagy of ESCC cells by functioning as miR-1299 sponge to target EGFR signaling. J Cell Biochem. (2019) 121:1039–49. doi: 10.1002/jcb.29339, PMID: 31490018

[62] Liu Y Lan S Duan Z. Circ-TTC17 promotes esophagus squamous cell carcinoma cell growth, metastasis, and inhibits autophagy-mediated radiosensitivity through miR-145-5p/SIRT1 axis. Thorac Cancer. (2024) 16:e15494. doi: 10.1111/1759-7714.15494, PMID: 39621506 PMC11729993

[63] Lin S Zhuang J Zhu L Jiang Z. Matrine inhibits cell growth, migration, invasion and promotes autophagy in hepatocellular carcinoma by regulation of circ_0027345/miR-345-5p/HOXD3 axis. Cancer Cell Int. (2020) 20:246. doi: 10.1186/s12935-020-01293-w, PMID: 32549793 PMC7296946

[64] Fu D Ji Q Wang C Yu L Yu R. Aloin decelerates the progression of hepatocellular carcinoma through circ_0011385/miR-149-5p/WT1 axis. Cell Cycle. (2021) 20:2476–93. doi: 10.1080/15384101.2021.1988227, PMID: 34720052 PMC8794511

[65] Zhao Z He J Feng C. CircCBFB is a mediator of hepatocellular carcinoma cell autophagy and proliferation through miR-424-5p/ATG14 axis. Immunol Res. (2022) 70:341–53. doi: 10.1007/s12026-021-09255-8, PMID: 35066780

[66] Wang X Dong FL Wang YQ Wei HL Li T Li J. Exosomal circTGFBR2 promotes hepatocellular carcinoma progression via enhancing ATG5 mediated protective autophagy. Cell Death Dis. (2023) 14:451. doi: 10.1038/s41419-023-05989-5, PMID: 37474520 PMC10359294

[67] Yang T Shen P Chen Q Wu P Yuan H Ge W. FUS-induced circRHOBTB3 facilitates cell proliferation via miR-600/NACC1 mediated autophagy response in pancreatic ductal adenocarcinoma. J Exp Clin Cancer Res. (2021) 40:261. doi: 10.1186/s13046-021-02063-w, PMID: 34416910 PMC8377879

[68] He Z Cai K Zeng Z Lei S Cao W Li X. Autophagy-associated circRNA circATG7 facilitates autophagy and promotes pancreatic cancer progression. Cell Death Dis. (2022) 13:233. doi: 10.1038/s41419-022-04677-0, PMID: 35288538 PMC8921308

[69] Tang Z Li J Lu B Zhang X Yang L Qi Y. CircBIRC6 facilitates the Malignant progression via miR-488/GRIN2D-mediated CAV1-autophagy signal axis in gastric cancer. Pharmacol Res. (2024) 202:107127. doi: 10.1016/j.phrs.2024.107127, PMID: 38438090

[70] Peng L Sang H Wei S Li Y Jin D Zhu X. CircCUL2 regulates gastric cancer Malignant transformation and cisplatin resistance by modulating autophagy activation via miR-142-3p/ROCK2. Mol Cancer. (2020) 19:156. doi: 10.1186/s12943-020-01270-x, PMID: 33153478 PMC7643398

[71] Shang Z Luo Z Wang Y Liu Q Xin Y Zhang M. CircHIPK3 contributes to cisplatin resistance in gastric cancer by blocking autophagy-dependent ferroptosis. J Cell Physiol. (2023) 238:2407–24. doi: 10.1002/jcp.31093, PMID: 37566605

[72] Fang L Lv J Xuan Z Li B Li Z He Z. Circular CPM promotes chemoresistance of gastric cancer via activating PRKAA2-mediated autophagy. Clin Transl Med. (2022) 12:e708. doi: 10.1002/ctm2.708, PMID: 35075806 PMC8787023

[73] Luo M Deng X Chen Z Hu Y. Circular RNA circPOFUT1 enhances Malignant phenotypes and autophagy-associated chemoresistance via sequestrating miR-488-3p to activate the PLAG1-ATG12 axis in gastric cancer. Cell Death Dis. (2023) 14:10. doi: 10.1038/s41419-022-05506-0, PMID: 36624091 PMC9829716

[74] Yang J Zhang X Cao J Xu P Chen Z Wang S. Circular RNA UBE2Q2 promotes Malignant progression of gastric cancer by regulating signal transducer and activator of transcription 3-mediated autophagy and glycolysis. Cell Death Dis. (2021) 12:910. doi: 10.1038/s41419-021-04216-3, PMID: 34611143 PMC8492724

[75] Zhang W Yang Q Qian D Zhao K Tang C Ju S. Deregulation of circRNA hsa_circ_0009109 promotes tumor growth and initiates autophagy by sponging miR-544a-3p in gastric cancer. Gastroenterol Rep (Oxf). (2024) 12:goae008. doi: 10.1093/gastro/goae008, PMID: 38425655 PMC10902679

[76] Chen Y Liu H Zou J Cao G Li Y Xing C. Exosomal circ_0091741 promotes gastric cancer cell autophagy and chemoresistance via the miR-330-3p/TRIM14/Dvl2/Wnt/β-catenin axis. Hum Cell. (2022) 36:258–75. doi: 10.1007/s13577-022-00790-6, PMID: 36323918

[77] Sang H Zhang W Peng L Wei S Zhu X Huang K. Exosomal circRELL1 serves as a miR-637 sponge to modulate gastric cancer progression via regulating autophagy activation. Cell Death Dis. (2022) 13:56. doi: 10.1038/s41419-021-04364-6, PMID: 35027539 PMC8758736

[78] Yao W Guo P Mu Q Wang Y. Exosome-derived circ-PVT1 contributes to cisplatin resistance by regulating autophagy, invasion, and apoptosis via miR-30a-5p/YAP1 axis in gastric cancer cells. Cancer Biother Radiopharm. (2021) 36:347–59. doi: 10.1089/cbr.2020.3578, PMID: 32799541

[79] Wei G Chen X Ruan T Ma X Zhu X Wen W. Human gastric cancer progression and stabilization of ATG2B through RNF5 binding facilitated by autophagy-associated CircDHX8. Cell Death Dis. (2024) 15:410. doi: 10.1038/s41419-024-06782-8, PMID: 38866787 PMC11169566

[80] Yang BL Liu GQ Li P Li XH. Circular RNA CUL2 regulates the development of colorectal cancer by modulating apoptosis and autophagy via miR-208a-3p-PPP6C. Aging (Albany NY). (2022) 14:497–508. doi: 10.18632/aging.203827, PMID: 35027503 PMC8791207

[81] Chen F Guo L Di J Li M Dong D Pei D. Circular RNA ubiquitin-associated protein 2 enhances autophagy and promotes colorectal cancer progression and metastasis via miR-582-5p/FOXO1 signaling. J Genet Genomics. (2021) 48:1091–103. doi: 10.1016/j.jgg.2021.07.017, PMID: 34416339

[82] Mo Y Lu Q Zhang Q Chen J Deng Y Zhang K. Circular RNA CCDC66 improves murine double minute 4 (MDM4) expression through targeting miR-370 in colorectal cancer. Comput Math Methods Med. (2022) 2022:7723995. doi: 10.1155/2022/7723995, PMID: 35069793 PMC8767369

[83] Pan Z Zheng J Zhang J Lin J Lai J Lyu Z. A novel protein encoded by exosomal circATG4B induces oxaliplatin resistance in colorectal cancer by promoting autophagy. Adv Sci (Weinh). (2022) 9:e2204513. doi: 10.1002/advs.202204513, PMID: 36285810 PMC9762280

[84] Yin D Zhai X Feng X Hua M Liu J Chen Y. Circ_0060927 promotes colorectal cancer development by sponging miR-331-3p and upregulating TBX2. Pathol Res Pract. (2024) 264:155673. doi: 10.1016/j.prp.2024.155673, PMID: 39486250

[85] Zhang Y Li C Liu X Wang Y Zhao R Yang Y. CircHIPK3 promotes oxaliplatin-resistance in colorectal cancer through autophagy by sponging miR-637. EBioMedicine. (2019) 48:277–88. doi: 10.1016/j.ebiom.2019.09.051, PMID: 31631038 PMC6838436

[86] Wang D Li Y Chang W Feng M Yang Y Zhu X. CircSEC24B activates autophagy and induces chemoresistance of colorectal cancer via OTUB1-mediated deubiquitination of SRPX2. Cell Death Dis. (2024) 15:693. doi: 10.1038/s41419-024-07057-y, PMID: 39333496 PMC11436887

[87] Sun J Wu H Luo J Qiu Y Li Y Xu Y. CircTBC1D22A inhibits the progression of colorectal cancer through autophagy regulated via miR-1825/ATG14 axis. iScience. (2024) 27:109168. doi: 10.1016/j.isci.2024.109168, PMID: 38439965 PMC10910227

[88] Weng W Wei Q Toden S Yoshida K Nagasaka T Fujiwara T. Circular RNA ciRS-7-a promising prognostic biomarker and a potential therapeutic target in colorectal cancer. Clin Cancer Res. (2017) 23:3918–28. doi: 10.1158/1078-0432.CCR-16-2541, PMID: 28174233 PMC5511556

[89] Mao W Wang K Xu B Zhang H Sun S Hu Q. CiRS-7 is a prognostic biomarker and potential gene therapy target for renal cell carcinoma. Mol Cancer. (2021) 20:142. doi: 10.1186/s12943-021-01443-2, PMID: 34740354 PMC8570002

[90] Pan H Li T Jiang Y Pan C Ding Y Huang Z. Overexpression of circular RNA ciRS-7 abrogates the tumor suppressive effect of miR-7 on gastric cancer via PTEN/PI3K/AKT signaling pathway. J Cell Biochem. (2017) 119:440–6. doi: 10.1002/jcb.26201, PMID: 28608528

[91] Liu L Liu FB Huang M Xie K Xie QS Liu CH. Circular RNA ciRS-7 promotes the proliferation and metastasis of pancreatic cancer by regulating miR-7-mediated EGFR/STAT3 signaling pathway. Hepatobil Pancreat Dis Int. (2019) 18:580–6. doi: 10.1016/j.hbpd.2019.03.003, PMID: 30898507

[92] Wang Q Zhang Q Sun H Tang W Yang L Xu Z. Circ-TTC17 promotes proliferation and migration of esophageal squamous cell carcinoma. Dig Dis Sci. (2018) 64:751–8. doi: 10.1007/s10620-018-5382-z, PMID: 30519852 PMC6394574

[93] Sun S Wang W Luo X Li Y Liu B Li X. Circular RNA circ-ADD3 inhibits hepatocellular carcinoma metastasis through facilitating EZH2 degradation via CDK1-mediated ubiquitination. Am J Cancer Res. (2019) 9:1695–707., PMID: 31497351 PMC6726993

[94] Mou T Xie F Zhong P Hua H Lai L Yang Q. MiR-345-5p functions as a tumor suppressor in pancreatic cancer by directly targeting CCL8. BioMed Pharmacother. (2019) 111:891–900. doi: 10.1016/j.biopha.2018.12.121, PMID: 30841468

[95] Zhang J Wang C Yan S Yang Y Zhang X Guo W. MiR-345 inhibits migration and stem-like cell phenotype in gastric cancer via inactivation of Rac1 by targeting EPS8. Acta Biochim Biophys Sin (Shanghai). (2020) 52:259–67. doi: 10.1093/abbs/gmz166, PMID: 32147678

[96] Zhou QY Gui SY Zhang P Wang M. Upregulation of miR-345-5p suppresses cell growth of lung adenocarcinoma by regulating ras homolog family member A (RhoA) and Rho/Rho associated protein kinase (Rho/ROCK) pathway. Chin Med J (Engl). (2021) 134:2619–28. doi: 10.1097/CM9.0000000000001804, PMID: 34748526 PMC8577671

[97] Zhang H Liu H Bi H. MicroRNA-345 inhibits hepatocellular carcinoma metastasis by inhibiting YAP1. Oncol Rep. (2017) 38:843–9. doi: 10.3892/or.2025.8913, PMID: 28677785 PMC5562085

[98] Chen F Pan Y Xu J Liu B Song H. Research progress of matrine’s anticancer activity and its molecular mechanism. J Ethnopharmacol. (2022) 286:114914. doi: 10.1016/j.jep.2021.114914, PMID: 34919987

[99] Ni C Yang S Ji Y Duan Y Yang W Yang X. Hsa_circ_0011385 knockdown represses cell proliferation in hepatocellular carcinoma. Cell Death Discov. (2021) 7:270. doi: 10.1038/s41420-021-00664-0, PMID: 34599150 PMC8486831

[100] Xia L Wu L Bao J Li Q Chen X Xia H. Circular RNA circ-CBFB promotes proliferation and inhibits apoptosis in chronic lymphocytic leukemia through regulating miR-607/FZD3/Wnt/β-catenin pathway. Biochem Biophys Res Commun. (2018) 503:385–90. doi: 10.1016/j.bbrc.2018.06.045, PMID: 29902450

[101] Yue J Zhu T Yang J Si Y Xu X Fang Y. CircCBFB-mediated miR-28-5p facilitates abdominal aortic aneurysm via LYPD3 and GRIA4. Life Sci. (2020) 253:117533. doi: 10.1016/j.lfs.2020.117533, PMID: 32151690

[102] Guan Z Tan J Gao W Li X Yang Y Li X. Circular RNA hsa_circ_0016788 regulates hepatocellular carcinoma tumorigenesis through miR-486/CDK4 pathway. J Cell Physiol. (2018) 234:500–8. doi: 10.1002/jcp.26612, PMID: 29923236

[103] Li W Lu H Wang H Ning X Liu Q Zhang H. Circular RNA TGFBR2 acts as a ceRNA to suppress nasopharyngeal carcinoma progression by sponging miR-107. Cancer Lett. (2021) 499:301–13. doi: 10.1016/j.canlet.2020.11.001, PMID: 33160003

[104] Espinosa EJ Calero M Sridevi K Pfeffer SR. RhoBTB3: a Rho GTPase-family ATPase required for endosome to golgi transport. Cell. (2009) 137:938–48. doi: 10.1016/j.cell.2009.03.043, PMID: 19490898 PMC2801561

[105] Matthys A Van Craenenbroeck K Lintermans B Haegeman G Vanhoenacker P. RhoBTB3 interacts with the 5-HT7a receptor and inhibits its proteasomal degradation. Cell Signal. (2012) 24:1053–63. doi: 10.1016/j.cellsig.2011.12.027, PMID: 22245496

[106] Long M Kranjc T Mysior MM Simpson JC. RNA interference screening identifies novel roles for RhoBTB1 and RhoBTB3 in membrane trafficking events in mammalian cells. Cells. (2020) 9:1089. doi: 10.3390/cells9051089, PMID: 32354068 PMC7291084

[107] Hu G Zhai S Yu S Huang Z Gao R. Circular RNA circRHOBTB3 is downregulated in hepatocellular carcinoma and suppresses cell proliferation by inhibiting miR-18a maturation. Infect Agent Cancer. (2021) 16:48. doi: 10.1186/s13027-021-00384-1, PMID: 34187598 PMC8243428

[108] Li X He S Ma B. Autophagy and autophagy-related proteins in cancer. Mol Cancer. (2020) 19:12. doi: 10.1186/s12943-020-1138-4, PMID: 31969156 PMC6975070

[109] Agrotis A Ketteler R. On ATG4B as drug target for treatment of solid tumours-the knowns and the unknowns. Cells. (2019) 9:53. doi: 10.3390/cells9010053, PMID: 31878323 PMC7016753

[110] Kristensen LS Jakobsen T Hager H Kjems J. The emerging roles of circRNAs in cancer and oncology. Nat Rev Clin Oncol. (2021) 19:188–206. doi: 10.1038/s41571-021-00585-y, PMID: 34912049

[111] Hao T Wang Z Yang J Zhang Y Shang Y Sun J. MALAT1 knockdown inhibits prostate cancer progression by regulating miR-140/BIRC6 axis. BioMed Pharmacother. (2020) 123:109666. doi: 10.1016/j.biopha.2019.109666, PMID: 31935634

[112] Luk ISU Shrestha R Xue H Wang Y Zhang F Lin D. BIRC6 targeting as potential therapy for advanced, enzalutamide-resistant prostate cancer. Clin Cancer Res. (2017) 23:1542–51. doi: 10.1158/1078-0432.CCR-16-0718, PMID: 27663589

[113] Yang H Zhao M Zhao L Li P Duan Y Li G. CircRNA BIRC6 promotes non-small cell lung cancer cell progression by sponging microRNA-145. Cell Oncol (Dordr). (2020) 43:477–88. doi: 10.1007/s13402-020-00503-x, PMID: 32297303 PMC12990671

[114] PuJYanXZhangH. The potential of circular RNAs as biomarkers and therapeutic targets for gastric cancer: A comprehensive review. J Adv Res. (2024), S2090–1232(24)00551-4. doi: 10.1016/j.jare.2024.11.032, PMID: 39617262

[115] Chen H Li F Xue Q. Circ-CUL2/microRNA-888-5p/RB1CC1 axis participates in cisplatin resistance in NSCLC via repressing cell advancement. Bioengineered. (2022) 13:2826–38. doi: 10.1080/21655979.2021.2024395, PMID: 35068326 PMC8974128

[116] Wang Y Cao Z Wang L Liu S Cai J. Downregulation of microRNA−142−3p and its tumor suppressor role in gastric cancer. Oncol Lett. (2018) 15:8172–80. doi: 10.3892/ol.2018.8330, PMID: 29849811 PMC5962855

[117] Wen J Liao J Liang J Chen XP Zhang B Chu L. Circular RNA HIPK3: a key circular RNA in a variety of human cancers. Front Oncol. (2020) 10:773. doi: 10.3389/fonc.2020.00773, PMID: 32500032 PMC7242753

[118] He S Luo Y Ma W Wang X Yan C Hao W. Endothelial POFUT1 controls injury-induced liver fibrosis by repressing fibrinogen synthesis. J Hepatol. (2024) 81:135–48. doi: 10.1016/j.jhep.2024.02.032, PMID: 38460791

[119] Long F Tian B Li L Ma M Chen Z Tan G. CircPOFUT1 fosters colorectal cancer metastasis and chemoresistance via decoying miR-653-5p/E2F7/WDR66 axis and stabilizing BMI1. iScience. (2024) 27:108729. doi: 10.1016/j.isci.2023.108729, PMID: 38230259 PMC10790033

[120] Yokota S Ogawara K Kimura R Shimizu F Baba T Minakawa Y. Protein O-fucosyltransferase 1: a potential diagnostic marker and therapeutic target for human oral cancer. Int J Oncol. (2013) 43:1864–70. doi: 10.3892/ijo.2013.2110, PMID: 24064921

[121] Dong S Wang Z Xiong W. POFUT1 promotes gastric cancer progression through Notch/Wnt dual signaling pathways dependent on the parafibromin-NICD1-β-catenin complex. J Chin Med Assoc. (2023) 86:806–17. doi: 10.1097/JCMA.0000000000000957, PMID: 37501238 PMC12755684

[122] Yu X Fu X Zhang X Bai C Wang Y. Circ_0001658 regulates gefitinib resistance of non-small cell lung cancer through miR-409-3p/TWIST1 axis. Anticancer Drugs. (2021) 33:158–66. doi: 10.1097/CAD.0000000000001257, PMID: 34694278

[123] Wang L Wang P Su X Zhao B. Circ_0001658 promotes the proliferation and metastasis of osteosarcoma cells via regulating miR-382-5p/YB-1 axis. Cell Biochem Funct. (2019) 38:77–86. doi: 10.1002/cbf.3452, PMID: 31758574

[124] Yu H Lee H Herrmann A Buettner R Jove R. Revisiting STAT3 signalling in cancer: new and unexpected biological functions. Nat Rev Cancer. (2014) 14:736–46. doi: 10.1038/nrc3818, PMID: 25342631

[125] You L Wang Z Li H Shou J Jing Z Xie J. The role of STAT3 in autophagy. Autophagy. (2015) 11:729–39. doi: 10.1080/15548627.2015.1017192, PMID: 25951043 PMC4509450

[126] Zhou Q Tian W Jiang Z Huang T Ge C Liu T. A positive feedback loop of AKR1C3-mediated activation of NF-κB and STAT3 facilitates proliferation and metastasis in hepatocellular carcinoma. Cancer Res. (2021) 81:1361–74. doi: 10.1158/0008-5472.CAN-20-2480, PMID: 33361392

[127] Kwan YP Teo MHY Lim JCW Tan MS Rosellinny G Wahli W. LRG1 promotes metastatic dissemination of melanoma through regulating EGFR/STAT3 signalling. Cancers (Basel). (2021) 13:3279. doi: 10.3390/cancers13133279, PMID: 34208965 PMC8269286

[128] Chen RY Yen CJ Liu YW Guo CG Weng CY Lai CH. CPAP promotes angiogenesis and metastasis by enhancing STAT3 activity. Cell Death Differ. (2019) 27:1259–73. doi: 10.1038/s41418-019-0413-7, PMID: 31511651 PMC7206147

[129] Wang F Ruan L Yang J Zhao Q Wei W. TRIM14 promotes the migration and invasion of gastric cancer by regulating epithelial−to−mesenchymal transition via activation of AKT signaling regulated by miR−195−5p. Oncol Rep. (2018) 40:3273–84. doi: 10.3892/or.2018.6750, PMID: 30272351 PMC6196628

[130] Ji XD Li G Feng YX Zhao JS Li JJ Sun ZJ. EphB3 is overexpressed in non-small-cell lung cancer and promotes tumor metastasis by enhancing cell survival and migration. Cancer Res. (2011) 71:1156–66. doi: 10.1158/0008-5472.CAN-10-0717, PMID: 21266352

[131] Liu C Li P Zheng J Wang Y Wu W Liu X. Role of necroptosis in airflow limitation in chronic obstructive pulmonary disease: focus on small-airway disease and emphysema. Cell Death Discov. (2022) 8:363. doi: 10.1038/s41420-022-01154-7, PMID: 35973987 PMC9381515

[132] Palcau AC Canu V Donzelli S Strano S Pulito C Blandino G. CircPVT1: a pivotal circular node intersecting long non-coding-PVT1 and c-MYC oncogenic signals. Mol Cancer. (2022) 21:33. doi: 10.1186/s12943-022-01514-y, PMID: 35090471 PMC8796571

[133] Zhang Q Tian H Ge K Wang F Gao P Chen A. PGD2/PTGDR2 signaling pathway affects the self-renewal capacity of gastric cancer stem cells by regulating ATG4B ubiquitination. Front Oncol. (2024) 14:1496050. doi: 10.3389/fonc.2024.1496050, PMID: 39777337 PMC11703842

[134] Wang H Liu S Sha X Gao X Liu G Jiang X. Unveiling the prominent roles of circular RNAs ubiquitin binding associated protein 2 in cancers. Pathol Res Pract. (2023) 241:154282. doi: 10.1016/j.prp.2022.154282, PMID: 36580797

[135] Hsiao KY Lin YC Gupta SK Chang N Yen L Sun HS. Noncoding effects of circular RNA CCDC66 promote colon cancer growth and metastasis. Cancer Res. (2017) 77:2339–50. doi: 10.1158/0008-5472.CAN-16-1883, PMID: 28249903 PMC5910173

[136] Feng J Li Z Li L Xie H Lu Q He X. Hypoxia−induced circCCDC66 promotes the tumorigenesis of colorectal cancer via the miR−3140-autophagy pathway. Int J Mol Med. (2020) 46:1973–82. doi: 10.3892/ijmm.2020.4747, PMID: 33125087 PMC7595663

[137] Chen J Xu L Fang M Xue Y Cheng Y Tang X. Hsa_circ_0060927 participates in the regulation of Caudatin on colorectal cancer Malignant progression by sponging miR-421/miR-195-5p. J Clin Lab Anal. (2022) 36:e24393. doi: 10.1002/jcla.24393, PMID: 35373390 PMC9102760

[138] Zhang Y Liu Q Liao Q. CircHIPK3 a promising cancer-related circular RNA. Am J Transl Res. (2020) 12:6694–704., PMID: 33194066 PMC7653572

[139] Zhang Z Yang T Xiao J. Circular RNAs: promising biomarkers for human diseases. EBioMedicine. (2018) 34:267–74. doi: 10.1016/j.ebiom.2018.07.036, PMID: 30078734 PMC6116471

[140] Ranieri R Ciaglia E Amodio G Picardi P Proto MC Gazzerro P. N6-isopentenyladenosine dual targeting of AMPK and Rab7 prenylation inhibits melanoma growth through the impairment of autophagic flux. Cell Death Differ. (2018) 25:353–67. doi: 10.1038/cdd.2017.165, PMID: 29027991 PMC5762849

[141] Jia L Meng Q Xu X. Autophagy-related miRNAs, exosomal miRNAs, and circRNAs in tumor progression and drug-and radiation resistance in colorectal cancer. Pathol Res Pract. (2024) 263:155597. doi: 10.1016/j.prp.2024.155597, PMID: 39426141

